# The WONE Index as a Multidimensional Assessment of Stress Resilience: A Development and Validation Study

**DOI:** 10.2196/81714

**Published:** 2026-01-05

**Authors:** Lydia Genevieve Roos, Destiny Gilliland, Kelsey Julian, Reeva Misra

**Affiliations:** 1 Walking on Earth, Ltd, International House 36-38 Cornhill, London, EC3V3NG, United Kingdom; 2 Department of Biobehavioral Health, College of Human Health and Development, The Pennsylvania State University, University Park, PA, United States; 3 Health Psychology PhD Program, College of Humanities and Earth and Social Sciences, University of North Carolina at Charlotte, Charlotte, NC, United States

**Keywords:** psychological stress, occupational stress, psychological resilience, occupational health, psychometrics, factor analysis, measurement validation, instrumentation

## Abstract

**Background:**

Stress resilience is a dynamic process shaped by the interaction between demands and adaptive resources. Existing measures assess stress and resilience as separate constructs, limiting their use in digital health and workplace interventions. An integrated measure capturing both domains is needed.

**Objective:**

We developed and validated the WONE Index, a multidimensional stress resilience tool designed to measure both current stress load and adaptive resources among full-time working adults.

**Methods:**

We developed the 32-item WONE Index through literature review, expert consultation, and iterative refinement to assess stress load and resilience resources across behavioral, cognitive, and social domains. Phase 1 (N=1005; United States– or United Kingdom–based full-time employees) evaluated the initial item pool using exploratory and confirmatory factor analyses to establish the preliminary factor structure and assess reliability and validity. Phase 2 (N=306; United States–based adults) expanded underperforming domains, refined items, and tested incremental validity, test-retest reliability, and measurement invariance. Data were collected online through CloudResearch (Connect) and Prolific (Prolific Academic Ltd) using secure survey platforms.

**Results:**

Phase 1 supported a 2-domain structure: a Stress Load factor (Work Stress, Personal Stress, and Burnout) and a Resilience Resources factor (Emotion Regulation and Coping, Social Connectedness, and Sleep). Model fit indices were excellent (comparative fit index, CFI=0.95; Tucker-Lewis index, TLI=0.94; and root mean square error of approximation, RMSEA=0.05). Phase 2 replicated and extended this structure, expanding Resilience Resources into 7 domains (adding Purpose and Prosociality, Physical Activity, Dietary Intake, and Perseverative Thinking). Confirmatory factor analyses supported a 2-domain structure, comprising a higher-order Stress Load factor with 3 subdomains (Work Stress, Personal Stress, and Burnout) and a higher-order Resilience Resources factor with 7 subdomains (Emotion Regulation and Coping, Social Connectedness, Purpose and Prosociality, Sleep, Physical Activity, Dietary Intake, and Perseverative Thinking). The Stress Load model demonstrated excellent fit (*χ*²_33_=64.18; *P*=.01; CFI=0.99; TLI=0.98; RMSEA=0.06; and standardized root mean square residual=0.05), and the Resilience Resources model also fit well (*χ*²_443_=745.20, *P*<.001; CFI=0.94; TLI=0.94; RMSEA=0.05; and standardized root mean square residual=0.06). All subscales showed strong internal consistency (composite reliability: mean 0.84, SD 0.10; range 0.74‐0.93) and excellent test-retest reliability over 3 weeks (intraclass correlation coefficients 0.77‐0.90, 95% CI 0.87-0.93). The index showed strong convergent validity (*r*=0.73 with Connor-Davidson Resilience Scale and *r*=–0.66 with Perceived Stress Scale-4) and explained additional variance beyond established measures in predicting depression, anxiety, and well-being (Δ*R*²=0.07‐0.11; *P*<.001).

**Conclusions:**

The WONE Index provides a psychometrically robust tool for assessing stress resilience capacity in working adults. Its integrated structure captures dynamic relationships between stress exposure and resilience resources, thereby supporting personalized intervention delivery in digital health platforms and organizational well-being programs.

## Introduction

### Background

Stress exposure is a pervasive challenge affecting individuals across work, personal, and societal domains, with well-documented impacts on psychological functioning, physical health, and quality of life [1,2]. While stress is common, individuals vary dramatically in their capacity to maintain well-being and adapt effectively under adversity—a quality captured by the concept of stress resilience [3-5]. As digital platforms increasingly seek to deliver personalized mental health support at scale, the ability to comprehensively assess individual resilience capacity has become critical [6,7]. However, existing resilience measures face fundamental limitations: they typically assess stress and resilience as separate constructs, provide insufficient detail to guide personalized interventions, and fail to capture the temporal dynamics through which resilience operates [8]. Addressing these measurement gaps is essential for advancing both resilience science and the development of effective digital mental health interventions across diverse contexts.

### Resilience as a Dynamic, Multifaceted Process

We define stress resilience as the dynamic capacity to effectively respond to, recover from, and adapt in the face of adversity while maintaining psychological well-being and functional capacity [9-11]. Rather than reducing resilience to a fixed trait or the simple absence of pathology, this view conceptualizes it as an active and multifaceted process [9]. This process emerges from the interaction between current stress exposure and available protective resources or vulnerabilities, spanning cognitive, behavioral, social, affective, and physiological domains [4,5].

### Job Demands-Resources Framework

This conceptualization aligns with the well-established job demands-resources (JD-R) model, which posits that psychological well-being results from the balance between demands that require sustained effort and resources that help manage those demands [12,13]. While originally developed for occupational contexts, this framework has been successfully extended to general stress and resilience processes, demonstrating that the demands-resources balance operates across life domains [14].

When demands exceed available resources, individuals experience strain and potential burnout. When resources are sufficient to meet demands, individuals can maintain well-being and even experience growth. This framework recognizes that resilience capacity is fundamentally determined by 2 interrelated but distinct domains: Stress Load (current psychological demands that require sustained effort) and Adaptive Resilience Resources (capacities that buffer demands and facilitate recovery).

Resilience Resources encompass the behavioral, cognitive, affective, and social capacities that enable adaptive responding to demands and facilitate recovery from adversity. These include emotion regulation skills, social support networks, physical health behaviors, cognitive flexibility, and meaning-making abilities. These resources function as protective factors against stress-related impairment [11]. Critically, many of these resources are modifiable through intervention, making them valuable targets for resilience-building efforts.

Having established the resource component of the JD-R framework, we now turn to the demands side of this balance, focusing on perceived stress. The JD-R framework aligns closely with the transactional model of stress [15-17], which emphasizes that the subjective appraisal of situational demands, rather than their objective frequency or intensity, is a strong determinant of psychological outcomes. We thus emphasize perceived stress rather than only objective stressors because individual appraisals of situational demands are more predictive of well-being and adaptation than event counts [16,17]. Within this perspective, cognitive appraisal serves as a regulatory mechanism that shapes whether a demand functions as a challenge that mobilizes engagement or as a hindrance that accelerates depletion.

The assessment of perceived stress presents both opportunities and challenges for resilience measurement. Perceived stress captures the subjectively experienced burden that directly impacts psychological functioning, making it more predictive of mental health outcomes than objective stressor inventories [18]. However, stress perceptions are themselves influenced by available resilience resources—individuals with stronger emotion regulation skills, social support, or coping strategies may appraise the same objective situation as less threatening [16,17]. This creates a dynamic relationship where resilience resources both protect against stress impact and influence stress perceptions themselves. Rather than viewing this complex relationship as a hindrance to accurate measurement, our framework recognizes this interdependence as fundamental to understanding stress resilience capacity.

Bringing these elements together, the demands-resources framework explains how stress resilience emerges from the interaction between these domains. High current demands deplete available psychological resources and can overwhelm coping capacity. On the other hand, strong resilience resources buffer against demand-related impact and facilitate faster recovery [14,19]. This dynamic interaction means that resilience capacity cannot be accurately assessed by measuring either demands or resources alone; comprehensive evaluation requires understanding both current demands (stress load) and available adaptive capacities (resilience resources).

However, a critical limitation remains in the classic JD-R model: it does not specify the temporal dynamics of these processes—the timescales over which demands erode resources or resources recover. This limitation is consequential for measurement. Because resilience represents an evolving balance rather than a fixed trait, capturing resilience capacity requires understanding both someone’s current demands and resources as well as how that balance changes over time. An individual experiencing high demands with adequate resources today may show progressive depletion over subsequent weeks, while another may maintain equilibrium or recover capacity despite similar initial states.

### The Need for New Measurement

The dynamic, interconnected nature of stress resilience processes creates significant measurement challenges. Traditional approaches that assess stress and resilience as separate, static constructs fail to capture the fundamental demands-resources interactions that determine resilience capacity [9]. This measurement gap is particularly problematic in digital mental health contexts. Assessment tools must simultaneously provide accurate evaluation of current psychological states and generate actionable insights for personalized intervention delivery.

Current measures have 3 critical limitations that prevent them from capturing these complex dynamics. First and most fundamentally, they fail to simultaneously assess both current perceived stress demands and available protective resources within the same framework. Traditional approaches measure either stress or resilience in isolation, missing the critical interplay between demands and adaptive capacities that determines whether individuals can maintain equilibrium, experience depletion, or build capacity. This separation makes it difficult to understand how an individual’s current psychological burden relates to their available coping capacities, limiting the ability to identify whether intervention should focus on stress reduction, resource building, or both.

Second, among measures that assess resilience, most are either too general or too narrow to guide personalized intervention. General measures, such as the Connor-Davidson Resilience Scale (CD-RISC [20]), effectively assess overall resilience characteristics but do not provide the detailed mapping of specific modifiable capacities—such as emotion regulation skills, social support quality, sleep patterns, or physical activity levels—that practitioners and digital platforms need to develop targeted intervention plans. Conversely, aspect-specific measures, such as the Brief Resilience Scale (BRS [21]), assess particular processes, such as stress recovery, effectively but do not capture the full range of behavioral, cognitive, and social factors that contribute to overall resilience capacity.

Third, existing measures do not adequately capture the temporal dynamics of resilience processes. As established earlier, resilience represents an evolving balance rather than a fixed trait, yet current measures typically provide only single-time-point snapshots rather than enabling tracking of how the balance between demands and resources changes over time.

Digital mental health platforms require assessment tools that address all 3 limitations by bridging the gap between comprehensive scientific measurement and practical intervention guidance. Such measures must simultaneously assess both stress and resources within a unified framework, provide sufficient detail to guide personalized interventions across multiple modifiable domains, and enable tracking of resilience trajectories over time through repeated administration. Critically, these tools must also be optimized for digital delivery contexts—brief enough for repeated mobile administration without user burden, structured to enable automated scoring and feedback, and designed to inform real-time algorithmic personalization of intervention content. The need for theoretically grounded measures meeting these criteria has become increasingly urgent as digital platforms seek to provide effective, scalable mental health interventions that address the dynamic nature of stress resilience capacity.

To address these measurement and intervention challenges, we developed the Walking on Earth (WONE; Walking on Earth, Ltd) Index as the assessment foundation for WONE, a digital stress resilience platform. Unlike traditional assessment tools developed for paper-and-pencil or clinical interview administration, the index was specifically designed for digital delivery via the WONE platform using mobile-optimized item presentation, response formats suited to digital interfaces, and brevity enabling repeated monthly assessment without user fatigue.

#### Two-Phase Validation Strategy

The validation of the WONE Index used a systematic 2-phase approach designed to address the complexity of developing a theoretically grounded yet multidimensional measure. This strategy allowed empirical findings from Phase 1 to inform the refinement of Phase 2.

Phase 1 served as an exploratory foundation study with four primary aims: (1) establishing the initial factor structure through traditional psychometric approaches, (2) identifying items and domains with adequate psychometric properties, (3) assessing convergent and criterion validity with established measures, and (4) revealing measurement challenges requiring refinement to inform Phase 2.

Phase 2 functioned as a confirmatory refinement phase with four key objectives: (1) implementing refined measurement models based on Phase 1 findings, (2) addressing identified measurement limitations through expanded item development, (3) establishing comprehensive psychometric properties, including temporal stability, and (4) demonstrating incremental validity beyond existing gold-standard measures.

## Methods

### Methods (Phase 1)

#### Participants

Participants (N=1005; 502 in the United States, and 503 in the United Kingdom) were recruited through CloudResearch (US sample) and Prolific (UK sample) using nonprobability quota sampling. Inclusion criteria included being aged 18‐65 years and working full-time (because the WONE user base targets adults working full-time), living in the United States or the United Kingdom (as the WONE platform currently operates primarily with companies based in these regions), and being fluent in English. We set quotas for gender within the CloudResearch and Prolific platforms to ensure adequate representation, such that neither men nor women are considered less than 45% of the study sample. Power analyses are provided in the “Data Analysis” section. Participants were balanced by gender (51% women in the United States and 48% in the United Kingdom), predominantly White (71% in the United States and 70% in the United Kingdom), with a mean age of 37.2 (SD 9.6) years in the United States and 36 (SD 10.4) years in the United Kingdom. Complete demographic characteristics for Phase 1 are provided in Table S1 in Multimedia Appendix 1 [22-24].

#### Procedures

All study procedures were conducted remotely using Qualtrics (Qualtrics, LLC) survey software. Participants accessed the survey through secure links distributed via CloudResearch (US sample) and Prolific (UK sample). After providing informed consent, participants completed the WONE Index and validation measures in a single sitting (approximately 20‐30 minutes). The survey was compatible with desktop and mobile devices. Built-in attention checks were included to ensure data quality. Participants who were unable to complete the survey in 1 session, except those who reported technical difficulties, or who did not pass at least 75% of attention checks, were disqualified. Participants were compensated for their time with US $6.25 or £4.50 for the US and UK samples, respectively.

#### Initial Item Development

The WONE Index item pool was developed through a targeted literature review and iterative expert consultation. We focused the literature review on identifying core constructs empirically linked to stress adaptation and resilience, drawing from theoretical and measurement work in stress appraisal, coping, self-regulation, social support, health behaviors, and well-being. We synthesized findings to determine the primary domains of adaptive capacity most consistently associated with mental health and performance outcomes.

Four health psychologists with expertise in stress and resilience then identified the constructs to be represented across both stress load and resilience resource domains, drawing on existing measures of perceived stress, burnout, resilience, coping, and health behaviors. In addition, we created new items to address domains not captured in previous tools, particularly those relevant to digital health and workplace contexts.

Through several collaborative meetings, the experts reviewed empirical evidence, debated conceptual boundaries, and progressively refined a broad initial pool into a smaller set of candidate items. Two specialists in digital health survey design then reviewed these items to evaluate clarity, readability, and redundancy for digital administration, after which additional consolidation produced the final 32-item instrument encompassing both current stress experiences and resilience resources.

Rather than imposing a fixed structure, the item pool was intentionally designed to allow empirical testing of whether stress load- and resource-related elements would remain distinct, overlap, or converge into higher-order domains. This theoretically grounded yet empirically flexible approach reflects our view of resilience as a dynamic, multidimensional construct rather than a static trait.

##### Current Stress Experiences

We tested 10 items to assess current stress load and burnout. For current stress, we assessed the following constructs: perceived stress, anxiety, and overwhelm at work and perceived stress, anxiety, and overwhelm at home/in one’s personal life. Our approach to stress measurement for perceived work and personal stress acknowledges that individuals conceptualize and express psychological strain differently in real-world contexts [25,26]. Rather than relying on a single “stress” indicator, we assessed 3 related but distinct constructs—stress, anxiety, and overwhelm—within both work and personal life domains.

Research demonstrates that individuals vary considerably in how they conceptualize and articulate psychological distress, with significant cultural and individual differences in whether experiences are described as “stress,” “anxiety,” “overwhelm,” or other terms [25,27]. Although academic literature often treats these as separate constructs, everyday users may not make such distinctions when describing their experiences [28]. Cultural, educational, and personal factors fundamentally influence how psychological experiences are articulated and understood [29,30].

For example, some individuals may more readily identify with feeling “overwhelmed” by their responsibilities, while others may describe similar experiences as “stress” or “anxiety.” By capturing all 3 dimensions, the WONE Index ensures a comprehensive assessment regardless of how someone naturally conceptualizes their distress, while also reflecting the interconnected nature of these stress-related experiences in daily life [31]. This inclusive measurement strategy maximizes the tool’s utility across diverse user populations and contexts.

We also included 4 indicators of burnout, which assessed cynicism, disengagement, reduced productivity, and mental exhaustion related to work, reflecting the core dimensions of occupational burnout as conceptualized in the Maslach Burnout Inventory framework [32,33]. These burnout indicators capture chronic workplace stress that differs qualitatively from acute stress experiences.

##### Resilience Resources

Resilience resources represent the psychological, social, and behavioral capacities that enable individuals to adapt to stress, recover from strain, and maintain well-being. This domain reflects the resources side of the WONE Index framework, balancing the stress-load indicators as described in the “Current Stress Experiences” section above.

Altogether, we tested 22 items to measure resilience resources. Nine emotion regulation and coping items consisted of emotion regulation, perceived ability to cope, tendency to bounce back after a stressor, adaptability, stress-related growth mindset, effective coping techniques, perspective-taking ability, frequency of positive affect, and perceived life meaning and purpose. Three self-efficacy and perceived control items assessed: self-efficacy beliefs, perceived control, and the sense that things were going smoothly. Two social support items assessed: trusted support availability and support satisfaction. Four sleep and energy items assessed: sleep quality, duration, latency, disturbances, and overall energy levels. Two dietary intake items included alcohol consumption and nutritious diet quality. Finally, 2 physical activity items assessed moderate and vigorous physical activity and sedentary time.

### Response Formats

Items used varying response formats optimized for each domain, all on 1‐5 point scales with domain-appropriate anchors, where higher numbers indicate better resilience. For example, response options included frequency scales (eg, “Never” to “Always”), agreement scales (eg, “Strongly Disagree” to “Strongly Agree”), and intensity scales (eg, “Not at all” to “Extremely”), selected based on item content and established measurement practices for each construct.

The WONE Index is organized into sections that pair construct-specific introductory instructions with domain-appropriate response formats. For example, stress-related items begin with “In the past month, how often have you felt…,” burnout items use agreement anchors, and health behavior items use categorical duration or quality scales. These variations are intentional, aligning response structure with the theoretical nature of each construct to ensure interpretability across diverse domains of stress and resilience. All items used a standardized 1-month timeframe (eg, “Over the past month...” or “In an average week over the past month…”) to ensure consistency, capture relatively stable patterns, and remain sensitive to change.

### External Validation Measures

Validated external scales were administered to assess criterion validity across stress, resilience, and mental health domains. These measures were used in both studies 1 and 2.

#### Stress Assessment

Perceived stress was assessed using the Perceived Stress Scale-4 (PSS-4 [18]), a brief version of one of the most widely used instruments for measuring stress perception [34]. The PSS-4 has demonstrated acceptable internal consistency (*α*=.60-.82 [34]) and good test-retest reliability over 4 weeks (*r*=0.73 [35]). It also shows strong convergent validity with measures of distress and discriminant validity from indicators of well-being [18]. The PSS-4 was selected for its brevity and suitability for rapid data collection while maintaining adequate psychometric robustness.

#### Resilience Assessment

Two established measures were used to assess resilience. The 10-item CD-RISC-10 [36] measures the ability to cope with adversity and demonstrates excellent internal consistency (*α*=.85-.92) as well as good convergent and discriminant validity across populations [36]. Although test-retest reliability was not re-examined for the 10-item version, the original 25-item CD-RISC shows strong temporal stability (*r*=0.87 [20]).

The 6-item BRS [21] measures the ability to recover or “bounce back” from stress and demonstrates high internal consistency (*α*=.80-.91), 1-month test-retest reliability (*r*=0.69), and robust convergent and discriminant validity with measures of affect, optimism, and neuroticism [21].

#### Mental Health and Well-being Outcomes

Depressive symptoms were measured using the Patient-Reported Outcomes Measurement Information System Short Form-8a (PROMIS-SF-8a [37]), which demonstrates excellent internal consistency (*α*>.90) and robust convergent validity with legacy measures such as the Patient Health Questionnaire-9 (PHQ-9) and Center for Epidemiologic Studies Depression (CES-D) [38] scales. Although test-retest data are not yet available for the fixed 8-item form, the PROMIS Depression computerized adaptive test shows high temporal stability (intraclass correlation coefficient [ICC]=0.86 [39]).

Anxiety was assessed with the Generalized Anxiety Disorder-7 (GAD-7 [40]), which has high internal consistency (*α*=.89-.92), good test-retest reliability (*r*=.83), and robust convergent and discriminant validity relative to other anxiety and depression scales [41].

Well-being was measured with the World Health Organization-5 Well-Being Index (WHO-5 [42]), which shows good internal consistency (*α*=.80-.90) and strong 5-day test-retest reliability (ICC=0.87 [43]), as well as robust convergent validity with measures of life satisfaction and positive affect [42].

### Data Analysis

#### Statistical Software

Statistical analyses were conducted using IBM SPSS Statistics (version 29.0). Confirmatory factor analyses were performed using IBM SPSS AMOS (version 22.0).

#### Data Quality, Screening, and Analytic Assumptions

Participants’ data were excluded from analyses if their responses to qualitative screening questions were nonsensical or irrelevant to the questions asked, as these were indicators of either automated responses or insufficient English fluency for valid participation. All survey items were required responses, resulting in no missing data for the final analytic sample.

Descriptive statistics and normality assessments were conducted for all key variables. Skewness and kurtosis values were all within the acceptable range of ±1.0, indicating reasonably symmetric distributions without problematic tail behavior. While Shapiro-Wilk tests indicated significant departures from normality for all variables (all *P*s<.001), this is expected given the large sample sizes, as these tests become overly sensitive to minor deviations with substantial samples [44]. Visual inspection of histograms and Q-Q plots confirmed that distributions were approximately normal with no severe violations.

Outlier analysis using boxplot methods identified a small number of extreme cases across variables (1‐11 outliers per variable, representing approximately 1%‐4% of cases). These outliers were retained as they represented valid responses within the expected range of the constructs and did not appear to result from data entry errors. Given the acceptable skewness and kurtosis values, robust nature of maximum likelihood estimation, and large sample sizes, the data were deemed appropriate for the planned factor analyses and correlational analyses.

#### Analytic Procedures

##### Exploratory Factor Analysis

Exploratory factor analysis (EFA) was conducted using principal axis factoring with Promax rotation to identify the underlying factor structure. Factor retention was determined using multiple criteria: (1) eigenvalues >1.0 (Kaiser criterion), (2) scree plot inspection, (3) theoretical interpretability, and (4) total variance explained. The Kaiser-Meyer-Olkin (KMO) measure of sampling adequacy and Bartlett test of sphericity were used to assess data appropriateness for factor analysis. We systematically evaluated items and retained them based on the following criteria: primary factor loadings ≥0.40, cross-loadings <0.30, difference between primary and secondary loadings ≥0.15, communalities ≥0.30, and theoretical coherence with factor interpretation.

##### Confirmatory Factor Analysis

We conducted a higher-order confirmatory factor analysis (CFA) using maximum likelihood estimation with robust SEs (Huber-White). The higher-order factors in the model consisted of latent factors related to (1) current stress experiences and (2) resilience resources. We evaluated model fit using multiple indices: *χ*²/df ratio (<5.0 acceptable and <3.0 good), comparative fit index (CFI ≥0.90 acceptable and ≥0.95 good), Tucker-Lewis index (TLI ≥0.90 acceptable and ≥0.95 good), root mean square error of approximation (RMSEA ≤0.08 acceptable and ≤0.06 good) and standardized root mean square residual (SRMR ≤0.08 acceptable and ≤0.06 good).

##### Reliability Assessment (Phase 1)

We assessed reliability using composite reliability (CR) estimates, which are more appropriate than Cronbach alpha for factor-based models, particularly in structural equation modeling frameworks [45]. CR was calculated using standardized factor loadings: CR=(Σλᵢ)²/[(Σλᵢ)² + Σ(1-λᵢ²)] [46]. Reliability ≥0.70 was considered acceptable for subscales and ≥0.90 for total scales.

##### Validity Testing (Phase 1)

Convergent validity was assessed through multiple approaches: (1) within the CFA by examining factor loading magnitudes (≥0.60 preferred and ≥0.40 minimum), statistical significance, and average variance extracted (AVE ≥0.50); and (2) through Pearson correlations with theoretically related established measures administered concurrently.

Discriminant validity was evaluated through multiple methods: (1) examining the CFA factor structure for cross-loadings and modification indices (MIs) suggesting misspecification, (2) computing the heterotrait-monotrait (HTMT) ratio with conservative (0.85) and liberal (0.90) thresholds, and (3) evaluating correlation patterns demonstrating stronger relationships between similar constructs than dissimilar constructs.

Concurrent validity was assessed using Pearson correlations with established measures administered simultaneously, specifically examining relationships between WONE Index scales and corresponding validated measures of the same constructs (eg, WONE Resilience Index with CD-RISC and BRS).

Criterion-related validity was evaluated by examining correlations between WONE Index scales and important mental health and well-being outcomes, including depression, anxiety, and psychological well-being.

##### Power Analysis

We conducted comprehensive a priori power analyses to determine the appropriateness of our targeted sample size of 1000 for the planned statistical tests, including factor analysis, reliability testing, validity assessment, and measurement invariance testing. We used a 2-tailed significance level of *α*=.05 for all analyses. Detailed power analyses are provided in Multimedia Appendix 1.

### Methods (Phase 2)

#### Participants and Procedure

We used the same nonprobability quota sampling recruitment strategy as Phase 1, except that we focused on US participants and recruited through CloudResearch. Participants were asked to complete surveys at 2 time points spaced 3 weeks apart. Participants were compensated US $5 at Time 1 and US $4 at Time 2; payment at Time 2 was slightly lower due to fewer items (eg, demographics). The analyses presented here focus on Time 1 data (N=306) for all EFAs and CFAs, with Time 2 used to assess test-retest reliability. Power analyses are provided within the “Data Analysis” section.

#### Theoretical Framework

Phase 2 was designed to refine and confirm the factor structure of the WONE Index based on the findings from Phase 1.

Phase 1 demonstrated that stress-related items formed a coherent, well-fitting factor structure, while several resilience domains—particularly health behaviors—required additional development. The strong validity evidence for the core stress and resilience skills components provided a solid foundation for expansion, while the challenges with nutrition, physical activity, and other behavioral domains indicated the need for larger item pools and more comprehensive domain coverage.

Phase 2 addresses these findings through several key design features. First, we substantially expanded the item pools for domains that showed promise but required development, particularly health behavior and social connection domains. Second, we adopted a domain-specific analytical approach, rather than the hierarchical approach used in Phase 1, allowing for comprehensive factor identification within each theoretical area while reducing model complexity. Third, we maintained the successful stress measurement structure from Phase 1 while expanding the resilience assessment to capture the full breadth of protective factors identified in resilience literature.

#### Domain Structure, Changes, and Measurement Strategy

Based on the findings and item performance from Phase 1, we adopted a staged, domain-specific approach that reflects both empirical patterns and theoretical considerations. The item pool was divided into 2 conceptually distinct domains for psychometric modeling and was analyzed within their respective models.

##### Resilience Resources Domain

Included items assessing behavioral and psychological capacities expected to support stress resilience. These spanned Emotion Regulation and Coping, Social Connectedness, Prosocial Values, Sleep, Physical Activity, and Dietary Intake. This comprehensive approach allowed for detailed examination of each resilience pathway while maintaining theoretical coherence across domains.

##### Stress Domain

We included the previously tested items under the Stress domain, reflecting stress experiences in one’s work and personal life, with strategic modifications based on Phase 1 findings and contemporary contextual factors. In addition, recognizing the chronic stress that has characterized daily life since the COVID-19 pandemic [47], including polarizing societal and political experiences that have become the norm within the past decade [48], global conflicts, and economic uncertainty, we added 1 item specifically assessing stress, anxiety, and overwhelm due to current societal, political, and economic issues. This addition acknowledges that contemporary stress experiences extend beyond traditional work and personal life domains to include broader societal stressors [49] that have become increasingly prominent in recent years.

### Measurement Strategy

Items were scored such that higher scores indicate greater stress or resilience resources when assessed separately, and the stress-load items were reverse-coded when assessed together so that higher overall scores reflected greater resilience. We analyzed both stress and resilience within their own respective models to reduce model complexity while still maintaining theoretical relevance. This staged approach is supported by previous psychometric validation practices, especially in multidimensional well-being assessments [50], and it allows for comprehensive development of each theoretical domain while maintaining the overarching framework that proved successful in Phase 1.

The domain-specific approach also identifies optimal factor structures for each area without the constraints imposed by simultaneous analysis of all domains. This approach is particularly important for health behavior domains that showed measurement challenges in Phase 1, allowing for detailed examination of their factor structure and item performance.

The WONE Index was developed and is copyrighted by Walking on Earth, Inc. The full administration materials, including detailed instructions and response options, are proprietary and therefore not publicly available. However, the measure may be available for use upon reasonable request to WONE via the website.

### External Validation Measures

The same validated external scales from Phase 1 were administered in Phase 2 to assess validity, with 2 additional measures included.

#### Depressive Symptoms

In addition to the PROMIS-SF-8a [37], which was used in Phase 1, we also administered the PHQ-8 [51], a modified version of the PHQ-9 that omits the ninth item on thoughts of death or self-harm. The PHQ-8 demonstrates high internal consistency (*α*=.82-.89 [51,52]) and strong short-term test-retest reliability (*r*=0.89 [52]). It also demonstrates diagnostic performance comparable to the PHQ-9, with nearly identical sensitivity, specificity, and overall accuracy [52,53]. We included this measure to enable comparison with the digital health literature that frequently uses PHQ-based instruments and to triangulate depression assessment using complementary approaches. While the PROMIS-SF-8a provides an abstract, mood-focused assessment with reduced somatic bias, the PHQ-8 offers a behaviorally anchored assessment directly mapping onto *DSM* (*Diagnostic and Statistical Manual of Mental Disorders*) criteria [51,54]. Together, the 2 instruments strengthen construct validation by capturing depressive symptoms from 2 distinct measurement approaches.

#### Personality Assessment

To assess discriminant validity, we included the brief Big Five Inventory (BFI-10 [55]). This short-form instrument measures extraversion, agreeableness, conscientiousness, neuroticism, and openness to experience, using 2 items per domain. The BFI-10 demonstrates acceptable internal consistency across traits (*α*=.65-.82), strong test-retest reliability (*r*=0.72-0.84), and high convergent validity with the full 44-item BFI (*r*=0.91-0.96 [55]).

### Data Quality, Screening, and Analytic Assumptions

Participants’ data were excluded from analyses if their responses to qualitative screening questions were nonsensical or irrelevant to the questions asked, as these were indicators of either automated responses or insufficient English fluency for valid participation. All survey items were required responses, resulting in no missing data for the final analytic samples (N=1005 for Phase 1 and N=306 for Phase 2).

Descriptive statistics and normality assessments were conducted for all key variables in both studies. Skewness and kurtosis values were all within the acceptable range of ±1.0 across both samples, indicating reasonably symmetric distributions without problematic tail behavior. Shapiro-Wilk tests indicated significant departures from normality for all variables in Phase 1 (all *P*<.001), which is expected given the large sample size. In Phase 2, normality tests were significant for external validation measures but not significant for the full WONE Index or Stress Subscale, with the Resilience Subscale showing marginal significance (*P*=.05). These patterns reflect the expected sensitivity of normality tests to sample size, with larger samples detecting minor deviations that are not practically meaningful [44]. Visual inspection of histograms and Q-Q plots confirmed that distributions were approximately normal with no severe violations.

Outlier analysis using boxplot methods identified minimal extreme cases: Phase 1 showed 1‐11 outliers per variable (representing 1%‐4% of cases), while Phase 2 showed substantially fewer outliers (0‐1 outliers per variable, representing <1% of cases). These outliers were retained as they represented valid responses within the expected range of the constructs and did not appear to result from data entry errors. Given the acceptable skewness and kurtosis values, the robust nature of maximum likelihood estimation, and adequate sample sizes, the data were deemed appropriate for the planned factor analyses and correlational analyses.

### Analytic Procedures

#### EFA

We used a staged EFA approach to identify the underlying factor structure within each domain. Principal axis factoring with Promax (oblique) rotation was used to accommodate the anticipated correlations between factors within each domain. For each EFA, sampling adequacy was assessed via the KMO statistic and Bartlett test of sphericity. Factor retention decisions were guided by eigenvalues >1.0, scree plot inspection, and theoretical coherence.

Items were retained if they met the following criteria: (1) primary loading ≥0.30, (2) minimal cross-loadings, and (3) thematic and theoretical fit with the emerging factor.

#### CFA

Following EFA, we specified and tested confirmatory factor models for each domain using maximum likelihood estimation with robust SEs. This domain-specific modeling strategy was selected to preserve theoretical clarity, allow for comprehensive factor development within each area, and build on the empirical structure observed in Phase 1, where resilience-related indicators and stress indicators showed distinct loading patterns.

##### Current Stress Model

Items loaded onto 3 first-order latent factors identified in Phase 1: Work Stress, Personal Stress, and Burnout. This model replicates the successful stress structure from Phase 1 while incorporating any additional stress-related items developed for Phase 2.

##### Resilience Resources Model

Items loaded onto 7 first-order latent factors representing expanded domains: Emotion Regulation and Coping, Social Connectedness, Purpose and Prosociality, Sleep, Physical Activity, Dietary Intake, and Perseverative Cognition. This expanded structure addresses the measurement challenges identified in Phase 1 by providing comprehensive coverage of resilience domains with sufficient items for stable factor identification.

The separate modeling approach allows for optimal factor structure identification within each domain while maintaining the theoretical framework established in Phase 1. Following successful domain-specific CFAs, the models can be integrated to test the overarching 2-factor higher-order structure (Stress and Resilience Resources) that demonstrated excellent validity in Phase 1. Model fit was evaluated using standard indices: CFI ≥0.90, RMSEA ≤0.08, and SRMR ≤0.08, consistent with conventional guidelines [56,57]. Model modifications were considered only when theoretically justifiable and indicated by MIs ≥10. When MIs suggested potential improvements, we prioritized residual covariances and factor-to-error covariances over cross-loadings to maintain interpretable factor structures while acknowledging theoretically expected relationships among constructs.

### Reliability Assessment (Phase 2)

Reliability assessment was performed using the same method as described above for phase 1.

### Validity Testing (Phase 2)

Convergent validity, concurrent validity, and criterion-related validity was assessed through multiple approaches, as described above for phase 1.

### Test-Retest Reliability

A subset of participants completed the WONE Index at a second time point approximately 3 weeks after their initial assessment to evaluate temporal stability. Test-retest reliability was assessed using ICC with a 2-way mixed-effects model for consistency of agreement. Single-measure ICCs (ICC[2,1]) were calculated for the full WONE Index, Stress Subscale, and Resilience Subscale. ICC values were interpreted using established guidelines: <0.50=poor, 0.50‐0.75=moderate, 0.75‐0.90=good, and >0.90=excellent reliability [58].

### Incremental Validity

Incremental validity was assessed through hierarchical multiple regression analyses to determine whether the WONE Index provides a meaningful prediction of mental health outcomes beyond established measures. Models tested incremental validity beyond: (1) the Perceived Stress Scale, (2) the CD-RISC, (3) the BRS, and (4) combined established measures. Mental health outcomes included depressive symptoms, anxiety symptoms, and well-being. Incremental validity was demonstrated by statistically significant R² change (ΔR²) when the WONE Index was added to models.

### Measurement Invariance Testing

We assessed measurement invariance of the Current Stress model using multigroup CFA, following the standard stepwise approach: configural, metric, scalar, and strict invariance. Each step imposed increasingly restrictive equality constraints across groups. Model fit was evaluated using commonly accepted thresholds for change in fit indices (ΔCFI ≤0.01, ΔRMSEA ≤0.015, and ΔSRMR ≤0.03 for metric invariance and ≤0.01 for scalar and strict invariance [59]). Fit was considered adequate when CFI and TLI were ≥0.95, RMSEA ≤0.06, and SRMR ≤0.08.

### Power Analysis

We conducted comprehensive a priori power analyses to determine the appropriateness of our targeted sample size of 300 for the planned statistical tests. We used a 2-tailed significance level of *α*=.05 for all analyses. Detailed power analyses are provided in Multimedia Appendix 1.

### Weighting Methodology

#### Hybrid Weighting Strategy Development

The WONE Index used a novel hybrid weighting approach that systematically integrates empirical prediction with theoretical importance and practical actionability. This methodology addresses a critical limitation in traditional psychometric approaches, in which purely data-driven weighting may undervalue theoretically important but empirically complex constructs, particularly in intervention-focused applications.

#### Empirical Weight Derivation

Empirical weights were derived through multiple regression analyses using each validation measure as a dependent variable and the 10 WONE factor scores as predictors. Standardized beta coefficients were extracted from regression models predicting 6 priority outcomes: CD-RISC-10, BRS, PSS-4, PHQ-8, GAD-7, and WHO-5.

Final empirical weights were calculated using a weighted average of standardized beta coefficients, with priority weights assigned based on theoretical importance: CD-RISC (25%), BRS (22%), PSS-4 (18%), PHQ-8 (15%), GAD-7 (12%), and WHO-5 (8%). For measures where higher scores indicate poorer outcomes (PSS-4, PHQ-8, and GAD-7), beta coefficients were reverse-scored to ensure all factors contributed positively to overall resilience scoring.

#### Theoretical Weight Framework

Theoretical weights were developed through a literature review [11,60-62] and author consensus, guided by the WONE Index’s core conceptual model emphasizing resilience capacity through perceived stress (resource depletion) and modifiable protective/risk factors (resource building/depletion). Theoretical weights were assigned based on three primary criteria: (1) strength of empirical evidence linking the construct to stress resilience outcomes, (2) theoretical importance within established resilience frameworks, and (3) behavioral actionability for intervention purposes.

#### Hybrid Integration and Final Weights

The final hybrid weights were calculated using a 50/50 integration formula: Final Weight = (Theoretical Weight×0.5) + (Empirical Weight×0.5). This approach preserves empirical predictive validity while ensuring meaningful representation of theoretically important and behaviorally actionable constructs. A minimum weight floor of 5% was applied to ensure all constructs maintain sufficient influence for generating actionable user recommendations in digital health application contexts. Constructs falling below this threshold after hybrid calculation were elevated to 5%, with proportional reductions applied to higher-weighted constructs to maintain a total weight sum of 100%.

### Ethical Considerations

This investigation was conducted in accordance with the Declaration of Helsinki and was determined to be exempt from Institutional Review Board (IRB) review after meeting ethical standards as evaluated by the Western Clinical Group IRB under 45 CFR § 46.104(d)(2) on November 27, 2024 (IRB ID 20244893). All participants provided informed consent before participation. Surveys were completed using SurveyMonkey (SurveyMonkey Inc), a GDPR-compliant platform with data encryption in transit and at rest, password-protected access controls, and secure data storage. Phase 1 participation was fully anonymous; no personally identifiable information was collected, and responses were recorded without linkage to any identifiers. In Phase 2, participant tracking across survey waves was managed through CloudResearch’s “Waves” feature, which assigns a randomly generated 32-character hexadecimal string as an identifier. This procedure enabled longitudinal matching of responses while preventing access to identifying information. Researchers did not have access to participants’ identities, and because surveys were administered in SurveyMonkey independently of CloudResearch, neither platform possessed both identity and response data. All data were stored on encrypted servers and analyzed in deidentified form.

## Results

### Phase 1: Results

#### Sample Demographics

Phase 1 included 1005 adults from the United States and the United Kingdom. Both samples were balanced by gender and predominantly White (refer to Table S1 in Multimedia Appendix 1).

#### Reliability of External Validation Measures

##### Stress Assessment

The PSS-4 demonstrated acceptable to good internal consistency, with reliability improving from Phase 1 (Cronbach *α*=0.79, 95% CI 0.77‐0.81; McDonald *ω*=0.84) to Phase 2 (Cronbach *α*=0.85, 95% CI 0.82‐0.88; McDonald *ω*=0.88). These reliability coefficients exceeded conventional thresholds for research use, supporting the measure’s suitability for criterion validity analyses.

##### Resilience Assessment

Both resilience measures exhibited excellent internal consistency across studies. The CD-RISC-10 maintained consistent reliability (Phase 1: Cronbach *α*=0.91, 95% CI 0.90‐0.92; McDonald *ω*=0.92 and Phase 2: Cronbach *α*=0.91, 95% CI 0.90‐0.93; McDonald *ω*=0.93), while the BRS improved from Phase 1 (Cronbach *α*=0.88, 95% CI 0.87‐0.90; McDonald *ω*=0.92) to Phase 2 (Cronbach *α*=0.92, 95% CI 0.91‐0.94; McDonald *ω*=0.94). The convergence between reliability indices confirms robust psychometric performance and establishes a strong foundation for resilience-related criterion validity.

##### Mental Health and Well-Being Outcomes

All mental health measures demonstrated exceptional reliability with coefficients consistently exceeding 0.90. The PROMIS Depression Short Form-8a achieved the highest consistency (Phase 1: Cronbach *α*=0.95, 95% CI 0.95‐0.96; McDonald *ω*=0.97 and Phase 2: Cronbach *α*=0.96, 95% CI 0.95‐0.97; McDonald *ω*=0.97), followed by the Generalized Anxiety Disorder-7 (Phase 1: Cronbach *α*=0.92, 95% CI 0.91‐0.93; McDonald *ω*=0.94 and Phase 2: Cronbach *α*=0.93, 95% CI 0.92‐0.94; McDonald *ω*=0.96) and WHO-5 (Phase 1: Cronbach *α*=0.91, 95% CI 0.90‐0.92; McDonald *ω*=0.93 and Phase 2: Cronbach *α*=0.92, 95% CI 0.91‐0.94; McDonald *ω*=0.92). These high reliability values ensure that criterion validity relationships reflect true associations rather than measurement error.

### EFA

Data demonstrated excellent suitability for factor analysis (KMO=0.926; Bartlett test: *χ*²_351_=13002.56; *P*<.001). Initially, 7 factors emerged—Resilience Skills and Beliefs, Work Stress, Personal Stress, Sleep, Burnout, Social Support, and Control—which accounted for 60.4% of the combined variance. Items related to alcohol, nutrition, sedentary time, and physical activity did not load onto any factor at ≥0.3, so they were removed. These findings indicated that health behavior domains required expanded item pools with more comprehensive measurement to achieve stable factor identification, which informed item development for Phase 2. The energy item loaded erroneously onto the Control factor and demonstrated a low Sleep loading, so it was also removed.

The final EFA with 27 items yielded a clear, interpretable 6-factor solution explaining 64.2% of the total variance: Resilience Skills and Beliefs, Work Stress, Personal Stress, Sleep, Burnout, and Social Support. Notably, the mental exhaustion item loaded onto the Work Stress factor instead of the Burnout factor. Although unexpected, mental exhaustion is typically the first symptom of burnout [63] and aligns conceptually with workplace stress; thus, the item was retained within the Work Stress factor. These items were then tested using the same factor placements in CFAs.

### CFA

A 2-factor higher-order CFA was conducted to test the proposed measurement model. The model specified Stress as a second-order latent construct comprising 3 first-order factors (Work Stress, Personal Stress, and Burnout) and Resilience Resources as a second-order latent construct comprising 3 first-order factors (Resilience Skills and Beliefs, Social Support, and Sleep Quality). The model included 33 observed indicators across 6 first-order factors. The initial higher-order model demonstrated adequate fit (*χ*²_317_=11,593.27; *P*<.001; CFI=0.90; TLI=0.89; RMSEA=0.07, 95% CI 0.06‐0.07; SRMR=0.058).

Following examination of MIs, residual covariances were freed for 15 conceptually related item pairs with MIs exceeding 20. The refined model achieved excellent fit (*χ*²_302_=993.30; *P*<.001; CFI=0.95; TLI=0.94; RMSEA=0.049, 95% CI 0.046‐0.052; SRMR=0.052). Although the chi-square test remained statistically significant—consistent with large sample size sensitivity [64]—all practical fit indices met or exceeded conventional standards for excellent model fit [56].

The Resilience Skills and Beliefs factor demonstrated strong overall performance, with loadings ranging from acceptable to strong (0.40≤ λ ≤0.76). Social Support emerged as the most cohesive first-order factor, with both indicators demonstrating exceptionally strong loadings (λ >0.80). Sleep showed a mixed but interpretable pattern, with 1 dominant indicator (sleep quality; λ=0.87) and 3 secondary indicators with more modest loadings (0.52≤ λ ≤0.63). The 3 stress factors (Work Stress, Personal Stress, and Burnout) all demonstrated strong psychometric properties (0.40≤ λ ≤0.76; refer to Table S3 in Multimedia Appendix 1).

### Second-Order Factor Structure

The higher-order structure received strong empirical support, with all second-order loadings exceeding 0.60 and demonstrating appropriate magnitudes. The Stress factor showed balanced contributions from its 3 constituent factors, with loadings ranging from 0.71 to 0.741, indicating that Work Stress, Personal Stress, and Burnout represent relatively equal contributors to the overarching stress construct.

Resilience Resources exhibited a more hierarchical structure, with Resilience Skills and Beliefs serving as the dominant indicator (λ=0.94) and Social Support and Sleep Quality contributing more modestly but substantially (λ=0.67 and 0.62, respectively). This pattern suggests that while all 3 resources are important components of resilience, individual resilience skills may serve as the core organizing feature of this higher-order construct.

### Reliability and Validity Testing

#### Internal Consistency Reliability

All factors demonstrated acceptable to excellent internal consistency reliability, as provided in Table S4 in Multimedia Appendix 1. CR estimates exceeded established thresholds across all factors. The full WONE Index showed excellent reliability, while subscale reliability was consistently strong across all first-order factors. All reliability estimates met or exceeded the 0.70 threshold for acceptable reliability, with most surpassing the 0.80 standard for good reliability.

#### Convergent Validity

The WONE Index demonstrated strong convergent validity with theoretically related established measures via correlations with externally validated measures. Resilience constructs showed substantial positive correlations with validated resilience measures: the Resilience Index correlated strongly with the CD-RISC (*r*=0.74; *P*<.001) and moderately with the BRS (*r*=0.68; *P*<.001). These correlations fall within the large effect size range [65], indicating that the WONE Resilience Index captures similar underlying resilience constructs while maintaining distinctiveness.

Stress constructs exhibited strong convergent validity with established stress and mental health measures. The Stress subscale correlated substantially with the PSS (*r*=0.66; *P*<.001), demonstrating convergent validity for the overall stress construct. Individual stress components showed expected relationships: positive correlations with depressive symptoms, anxiety symptoms, and perceived stress, as well as a negative correlation with well-being, were observed, supporting the validity of the stress measurement model.

AVE estimates, calculated as the mean of the communalities (R²) for each factor’s items (refer to Table S3 in Multimedia Appendix 1), provided evidence of convergent validity for most factors. Four factors met or exceeded the 0.50 criterion, while 2 factors showed AVE values below 0.50 but above 0.40. While AVE values below 0.50 suggest some concern for convergent validity, the strong CR estimates and substantial factor loadings discussed previously indicate that convergent validity is still adequate for these factors [46].

#### Discriminant Validity

HTMT analysis provided strong evidence for discriminant validity. All HTMT values fell well below the conservative 0.85 threshold, with an average value of 0.49, indicating excellent discriminant validity between all factor pairs (refer to Table S4 in Multimedia Appendix 1).

#### Concurrent Validity

The WONE Index demonstrated excellent concurrent validity with established criterion measures (refer to Table S5 in Multimedia Appendix 1). The Resilience subscale showed strong concurrent validity with both the CD-RISC and BRS, 2 well-validated resilience measures with different theoretical emphases. The Stress subscale demonstrated substantial concurrent validity with the PSS, a widely used measure of subjective stress appraisal.

#### Criterion-Related Validity

The WONE Index showed strong criterion-related validity with important mental health and well-being outcomes (refer to Table S5 in Multimedia Appendix 1). The full WONE Index demonstrated robust associations with all criterion measures, showing positive correlations with well-being and negative correlations with both depressive and anxiety symptoms.

The Stress subscale exhibited strong criterion-related validity, with negative associations with well-being and positive associations with both depressive and anxiety symptoms. The Resilience subscale demonstrated complementary criterion-related validity, with positive associations with well-being and negative associations with both depressive and anxiety symptoms.

### Phase 2 Results

#### Sample Demographics

Phase 2 included 306 US adults. The sample was balanced by gender and predominantly White (refer to Table S6 in Multimedia Appendix 1). Although the study was open to participants aged 18‐64 years, the youngest participant was aged 20 years, and the oldest was aged 63 years, resulting in an age range of 20‐63 years.

#### Reliability of External Validation Measures

##### Depressive Symptoms

The PHQ-8 demonstrated excellent internal consistency (Cronbach α=0.90, 95% CI 0.88-0.91; McDonald ω=0.93). This strong reliability supports its use as a complementary measure to the PROMIS-SF-8a and strengthens construct validation through convergent evidence across different measurement approaches.

##### Personality

Reliability for the BFI-10 subscales was modest, consistent with expectations for short-form personality measures. Extraversion (α=.71 and ω=0.71) and Neuroticism (α=.77 and ω=0.77) showed adequate reliability, while Conscientiousness (α=.58 and ω=0.63), Agreeableness (α=.57 and ω=0.57), and Openness (α=.58 and ω=0.63) were lower. These results are consistent with previous findings indicating that short-form personality measures often exhibit attenuated reliability due to their limited item coverage per construct.

### EFA Results

#### Stage 1: Resilience Factors Analysis

##### Data Suitability and Sample Characteristics

Analysis was conducted on 306 participants using 51 resilience-related items. Data demonstrated excellent suitability for factor analysis: KMO measure of sampling adequacy=0.91 (excellent), and Bartlett test of sphericity was highly significant (*χ*²_528_=5790.89; *P*<.001), confirming the appropriateness of the correlation matrix for factorization.

##### Factor Extraction and Retention Strategy

Based on theoretical considerations, we specified a maximum of 7 factors corresponding to hypothesized resilience domains: (1) Emotion Regulation and Coping Tendencies, (2) Resilience-Related Beliefs, (3) Positive Psychology Buffers, (4) Social Connectedness, (5) Dietary Intake, (6) Physical Activity, and (7) Sleep.

##### Item Reduction and Refinement Process

Through iterative analysis, we applied stringent psychometric criteria for item retention. Items were systematically removed based on (1) low communalities (<0.30), (2) inadequate factor loadings (<0.40), and (3) theoretically inappropriate cross-loadings. This process resulted in the removal of 15 items.

##### Final Factor Structure and Variance Explained

The final 7-factor solution with 33 items explained 66.04% of the total variance, demonstrating substantial explanatory power. Factor loadings ranged from 0.42 to 1.00, with the majority of items (26/33, 78.79%) exceeding 0.50, indicating strong factor-item relationships. Alternative solutions with 5 or 6 factors were examined but yielded weaker, less interpretable factor structures.

### Stage 2: Stress Factors EFA

#### Data Suitability and Factor Extraction

Analyses using 13 stress-related items with the same sample (N=306) demonstrated good data suitability (KMO=0.87; Bartlett test *χ*²_55_=2186.66; *P*<.001). Based on theoretical considerations and Phase 1 findings, we specified 3 stress factors corresponding to distinct stress domains, assuming that the new item related to societal, political, and economic stress would align with the Personal Stress factor. Principal axis factoring with Promax rotation converged in 6 iterations.

#### Factor Structure and Item Performance

The 3-factor solution explained 74.76% of total variance. Two items were removed due to substantial cross-loadings across stress domains: Perceived Control (“Felt that you have control over the important things in your life”) and Smooth (“That things were going smoothly for you”) both loaded significantly onto both Work Stress and Personal Stress factors, indicating these items captured general perceived control and life satisfaction rather than domain-specific stressors. The final 11 items demonstrated a clear factor structure with strong psychometric properties.

Notably, the exhaustion item loaded onto the Work Stress rather than the Burnout factor, which is consistent with theoretical models positioning exhaustion as an early indicator of work-related stress that may precede full burnout syndrome.

### Integrated Factor Structure Summary

The staged EFA approach successfully identified a robust 10-factor structure encompassing 43 items: 7 resilience factors (32 items) and 3 stress factors (11 items). This comprehensive framework captures both current stress exposure and modifiable factors that influence resilience capacity, providing a theoretically grounded and empirically supported foundation for comprehensive stress resilience assessment.

The excellent psychometric properties, substantial variance explained (66.04% for resilience factors and 74.76% for stress factors), and strong reliability estimates support the validity of this multidimensional approach to measuring stress resilience capacity in applied settings.

### CFA Results

#### Current Stress Model

We conducted a CFA to evaluate the structure of the Current Stress domain, which specified a higher-order latent factor (Stress) comprised of 3 latent factors: Work Stress, Personal Stress, and Burnout. The initial model demonstrated adequate fit to the data (*χ*²_41_=204.52; *P*<.001; CFI=0.92; TLI=0.90; RMSEA=0.11; and SRMR=0.07). We used MIs ≥10 to identify potential covariances between item residuals and added the following theoretically justified covariances to improve model fit without altering the core factor structure (refer to Multimedia Appendix 1).

These modifications reflected close semantic or contextual overlap among items and improved the overall model fit. Factor-to-error covariances were preferred over cross-loadings to maintain clear factor interpretation while acknowledging systematic relationships between theoretically related stress constructs.

The revised model demonstrated excellent fit to the data (*χ*²_33_=64.18; *P*=.001; CFI=0.99; TLI=0.98; RMSEA=0.06; and SRMR=0.05). All standardized factor loadings were statistically significant and strong, ranging from 0.54 to 0.92 across the 11 items. Most loadings exceeded 0.75, supporting the construct validity of the latent factors. Squared multiple correlations (R²) showed that most items explained a substantial proportion of variance, ranging from 0.29 to 0.84. Full standardized factor loadings, SEs, and communalities are provided in Table 1. The WONE Index is a proprietary measure copyrighted by Walking on Earth, Inc. Full administration materials are available upon reasonable request.

**Table 1.** Standardized factor loadings and communalities for Phase 2 confirmatory factor analysis (CFA) models.

Construct	Factor	Domain	Standardized loading^a^	SE	Communality (*R*²)
Stress (personal)	Personal Stress	Stress	0.92	—^b^	0.84
Anxiousness (personal)	Personal Stress	Stress	0.87	0.05	0.75
Overwhelm (personal)	Personal Stress	Stress	0.86	0.05	0.74
Societal, political, and economic stress	Personal Stress	Stress	0.54	0.06	0.29
Stress (work)	Work Stress	Stress	0.90	—	0.82
Anxiousness (work)	Work Stress	Stress	0.76	0.06	0.58
Overwhelm (work)	Work Stress	Stress	0.83	0.06	0.69
Mental exhaustion	Work Stress	Stress	0.79	0.07	0.63
Disengagement	Burnout	Stress	0.86	—	0.74
Cynicism	Burnout	Stress	0.75	0.08	0.56
Lack of productivity	Burnout	Stress	0.75	0.08	0.56
Vigorous physical activity	Physical Activity	Resilience Resources	0.99	0.11	1.00
Moderate physical activity	Physical Activity	Resilience Resources	0.63	—	0.40
Sleep quality	Sleep	Resilience Resources	0.85	—	0.72
Fatigue	Sleep	Resilience Resources	0.74	0.07	0.55
Sleep duration	Sleep	Resilience Resources	0.47	0.10	0.22
Sleep latency	Sleep	Resilience Resources	0.62	0.08	0.38
Sleep disturbances	Sleep	Resilience Resources	0.64	0.08	0.41
Nutritious food intake	Dietary Intake	Resilience Resources	0.65	—	0.42
Processed food intake	Dietary Intake	Resilience Resources	0.50	0.13	0.25
Caffeine intake	Dietary Intake	Resilience Resources	0.32	0.13	0.10
Caffeine reliance	Dietary Intake	Resilience Resources	0.52	0.19	0.27
Emotion regulation	Emotion Regulation and Coping	Resilience Resources	0.74	0.06	0.68
Emotional understanding	Emotion Regulation and Coping	Resilience Resources	0.61	0.06	0.37
Distress tolerance	Emotion Regulation and Coping	Resilience Resources	0.70	0.06	0.49
Acceptance	Emotion Regulation and Coping	Resilience Resources	0.50	0.06	0.25
Ability to bounce back	Emotion Regulation and Coping	Resilience Resources	0.76	0.06	0.58
Adaptability	Emotion Regulation and Coping	Resilience Resources	0.73	0.05	0.53
Effective coping	Emotion Regulation and Coping	Resilience Resources	0.83	—	0.69
Self-efficacy	Emotion Regulation and Coping	Resilience Resources	0.68	0.06	0.47
Cognitive flexibility	Emotion Regulation and Coping	Resilience Resources	0.66	0.06	0.43
Dwelling	Perseverative Thinking	Resilience Resources	0.91	—	0.82
Worrying	Perseverative Thinking	Resilience Resources	0.88	0.06	0.78
Meaning and purpose	Purpose and Prosociality	Resilience Resources	0.54	0.10	0.44
Gratitude	Purpose and Prosociality	Resilience Resources	0.78	0.09	0.61
Compassion for others	Purpose and Prosociality	Resilience Resources	0.63	0.09	0.40
Consideration for others	Purpose and Prosociality	Resilience Resources	0.67	0.07	0.45
Making a positive difference	Purpose and Prosociality	Resilience Resources	0.79	—	0.63
Trusted support system	Social Connection	Resilience Resources	0.89	0.05	0.79
Support satisfaction	Social Connection	Resilience Resources	0.87	0.06	0.76
Strength from close others	Social Connection	Resilience Resources	0.87	—	0.76
Belongingness	Social Connection	Resilience Resources	0.85	0.05	0.72
Loneliness	Social Connection	Resilience Resources	0.83	0.06	0.69

a All factor loadings are statistically significant at *P*<.001.

b SE not reported when factor loading was fixed to 1 for model identification.

#### Second-Order Factor Structure

The higher-order Stress factor was well supported by strong loadings from all 3 first-order factors. Work Stress showed the strongest loading (λ=0.80 and SE=0.12), followed by Personal Stress (λ=0.76) and Burnout (λ=0.67 and SE=0.13). The second-order loadings ranged from 0.67 to 0.80, with an average of 0.74, indicating that all 3 stress domains contribute substantially and relatively equally to the overarching stress construct. The squared multiple correlations for the first-order factors (0.44-0.65) demonstrate that the higher-order factor explains a substantial proportion of variance in each stress domain.

#### Resilience Resources Model

We then conducted a CFA to assess the structure of the Resilience Resources model, which specified a higher-order latent factor (Resources) comprised of 7 first-order latent domains: Emotion Regulation and Coping, Social Connectedness, Compassion and Gratitude, Sleep, Physical Activity, Dietary Intake, and Perseverative Thinking. The initial model demonstrated adequate fit to the data (*χ*²_458_=1081.71; *P*<.001; CFI=0.88; TLI=0.87; RMSEA=0.07; and SRMR=0.09). After reviewing MIs≥10, we added theoretically grounded modifications (refer to Multimedia Appendix 1 for specifics).

These modifications reflect meaningful psychological relationships among resilience constructs and improved model fit substantially while preserving the primary factor structure. This approach was chosen over alternative specifications (eg, cross-loadings) to maintain interpretability while acknowledging the theoretically expected interconnections among resilience domains.

The revised model fit the data well (*χ*²_443_=745.20; *P*<.001; CFI=0.94; TLI=0.94; RMSEA=0.05; and SRMR=0.06). All standardized factor loadings were statistically significant, ranging from 0.31 to 0.99. Most loadings exceeded 0.60, indicating strong relationships between latent constructs and their corresponding indicators. Squared multiple correlations (*R*²) demonstrated good explanatory power for most observed variables, supporting the reliability and coherence of the factor structure. The confirmed 10-factor structure represents the final, validated framework of the WONE Index. Each factor captures specific theoretical domains as described in the “Second-Order Factor Structure" section. Item-level psychometric details are provided in Table 1, and theoretical descriptions of the factors are included in the “Discussion” section for Phase 2.

#### Second-Order Factor Structure

The higher-order Resilience Resources factor showed a more varied pattern of loadings from the 7 first-order factors, ranging from 0.31 to 0.88 (average=0.59). Emotion Regulation and Coping emerged as the dominant contributor (λ=0.88), followed by Perseverative Thinking (λ=0.73) and Sleep (λ=0.78). Social Connection showed a moderate loading (λ=0.55), while Purpose and Prosociality (λ=0.37) and Physical Activity (λ=0.31) contributed more modestly to the overarching construct. This hierarchical pattern suggests that while multiple domains contribute to resilience resources, cognitive-emotional regulatory capacities serve as the primary organizing feature, with behavioral and social factors providing important but secondary contributions.

Although each domain was analyzed separately for model estimation and reporting, together these 2 validated higher-order structures conceptually represent a correlated higher-order framework consistent with JD-R theory. This framework conceptualizes Stress Load and Resilience Resources as distinct but interrelated systems that cannot be reduced to a single overarching factor. The correlated-factor interpretation was retained over a bifactor alternative because it better reflects the theoretical position that resilience emerges from dynamic interactions among multiple interdependent subsystems rather than from a single general dimension.

The 2 higher-order factors were also strongly correlated at the scale level, which indicates that higher stress load was statistically associated with lower resilience resources, although this relationship was not modeled within the same CFA structure. The squared multiple correlations for the first-order factors ranged from 0.10 to 0.78, indicating substantial variation in how well the higher-order factor explains variance across different resilience domains.

### Validity and Reliability

All correlations between the WONE Index and external measures are provided in Table 2.

**Table 2.** Phase 2 correlations between the Walking on Earth (WONE) Index and established measures.

Variable		Full WONE Index	WONE stress subscale	WONE resilience subscale	PSS^a^	CD-RISC^b^	BRS^c^	PHQ-8^d^	PROMIS-SF-8a^e^	GAD-7^f^	WHO-5^g^	BFI-E^h^	BFI-A^i^	BFI-C^j^	BFI-N^k^	BFI-O^l^
Full WONE Index																
	r	1	–0.84	0.98	–0.81	0.75	0.74	–0.77	–0.79	–0.77	0.83	0.31	0.46	0.45	–0.74	–0.03
	*P* value	—^m^	<.001	<.001	<.001	<.001	<.001	<.001	<.001	<.001	<.001	<.001	<.001	<.001	<.001	.56
WONE stress subscale																
	r	–0.84a	1	–0.71	0.73	–0.52	–0.57	0.69	0.71	0.75	–0.68	–0.21	–0.31	–0.39	0.66	0.03
	*P* value	<.001	—	<.001	<.001	<.001	<.001	<.001	<.001	<.001	<.001	<.001	<.001	<.001	<.001	.59
WONE resilience subscale																
	r	0.98a	–0.71	1	–0.78	0.77	0.75	–0.73	–0.76	–0.71	0.82	0.33	0.47	0.44	–0.71	–0.03
	*P* value	<.001	<.001	—	<.001	<.001	<.001	<.001	<.001	<.001	<.001	<.001	<.001	<.001	<.001	.58
PSS																
	r	–0.81	0.73	–0.78	1	–0.66	–0.68	0.72	0.80	0.76	–0.74	–0.26	–0.36	–0.41	0.61	0.02
	*P* value	<.001	<.001	<.001	—	<.001	<.001	<.001	<.001	<.001	<.001	<.001	<.001	<.001	<.001	.74
CD-RISC																
	r	0.75	–0.52	0.77	–0.66	1	0.83	–0.52	–0.57	–0.53	0.66	0.33	0.33	0.42	–0.65	–0.06
	*P* value	<.001	<.001	<.001	<.001	—	<.001	<.001	<.001	<.001	<.001	<.001	<.001	<.001	<.001	.27
BRS																
	r	0.74	–0.57	0.75	–0.68	0.83	1	–0.53	–0.57	–0.58	0.62	0.27	0.40	0.39	–0.68	–0.05
	*P* value	<.001	<.001	<.001	<.001	<.001	—	<.001	<.001	<.001	<.001	<.001	<.001	<.001	<.001	.44
PHQ-8																
	r	–0.77	0.69	–0.73	0.72	–0.52	–0.53	1	0.85	0.81	–0.75	–0.22	–0.34	–0.41	0.55	0.05
	*P* value	<.001	<.001	<.001	<.001	<.001	<.001	—	<.001	<.001	<.001	<.001	<.001	<.001	<.001	.36
PROMIS-SF-8a																
	r	–0.79	0.71	–0.76	0.80	–0.57	–0.57	0.85	1	0.78	–0.76	–0.29	–0.40	–0.41	0.55	0.04
	*P* value	<.001	<.001	<.001	<.001	<.001	<.001	<.001	—	<.001	<.001	<.001	<.001	<.001	<.001	.45
GAD-7																
	r	–0.77	0.75	–0.71	0.76	–0.53	–0.58	0.81	0.78	1	–0.67	–0.19	–0.30	–0.33	0.67	0.06
	*P* value	<.001	<.001	<.001	<.001	<.001	<.001	<.001	<.001	—	<.001	<.001	<.001	<.001	<.001	.27
WHO-5																
	r	0.83	–0.68	0.82	–0.74	0.66	0.62	–0.76	–0.75	–0.67	1	0.32	0.42	0.43	–0.61	–0.04
	*P* value	<.001	<.001	<.001	<.001	<.001	<.001	<.001	<.001	<.001	—	<.001	<.001	<.001	<.001	.53
BFI-E																
	r	0.31	–0.21	0.33	–0.26	0.33	0.27	–0.29	–0.22	–0.19	0.32	1	0.30	0.25	–0.33	0.02
	*P* value	<.001	<.001	<.001	<.001	<.001	<.001	<.001	<.001	<.001	<.001	—	<.001	<.001	<.001	.75
BFI-A																
	r	0.46a	–0.31	0.47	–0.34	0.33	0.40	–0.40	–0.35	–0.30	0.42	0.30	1	0.34	–0.33	–0.05
	*P* value	<.001	<.001	<.001	<.001	<.001	<.001	<.001	<.001	<.001	<.001	<.001	—	<.001	<.001	.34
BFI-C																
	r	0.45a	–0.39	0.44	–0.41	0.42	0.34	–0.41	–0.41	–0.33	0.43	0.25	0.34	1	–0.38	0.02
	*P* value	<.001	<.001	<.001	<.001	<.001	<.001	<.001	<.001	<.001	<.001	<.001	<.001	—	<.001	.76
BFI-N																
	r	–0.74	0.66	–0.71	0.61	–0.65	–0.68	0.55	0.55	0.67	–0.61	–0.33	–0.33	–0.38	1	0.02
	*P* value	<.001	<.001	<.001	<.001	<.001	<.001	<.001	<.001	<.001	<.001	<.001	<.001	<.001	—	.67
BFI-O																
	r	–0.03	0.03	–0.03	0.02	–0.06	–0.05	0.04	0.05	0.06	–0.04	0.02	–0.05	0.02	0.02	1
	*P* value	<.001	<.001	<.001	<.001	<.001	<.001	<.001	<.001	<.001	<.001	<.001	<.001	<.001	<.001	—

a PSS: Perceived Stress Scale.

b CD-RISC: Connor-Davidson Resilience Scale.

c BRS: Brief Resilience Scale.

d PHQ-8: Patient Health Questionnaire-8.

e PROMIS-SF-8a: Patient-Reported Outcomes Measurement Information System Short Form 8a.

f GAD-7: Generalized Anxiety Disorder-7.

g WHO-5: World Health Organization-5 Well-Being Index.

h BFI-E: Big Five Inventory-Extraversion

i BFI-A: Big Five Inventory-Agreeableness

j BFI-C: Big Five Inventory-Conscientiousness

k BFI-N: Big Five Inventory-Neuroticism

l BFI-O: Big Five Inventory-Openness

m Not applicable.

#### Construct-Level Convergent Validity

The WONE Index demonstrated strong construct-level convergent validity with established measures of stress and resilience. The Stress Subscale showed a large positive correlation with the PSS, indicating that higher scores on our stress measure align closely with higher perceived stress as measured by this gold-standard instrument.

For resilience measures, the Resilience Subscale demonstrated excellent convergent validity with both the CD-RISC-10 and the BRS. Similarly, the Full WONE Index showed strong positive correlations with both resilience measures. These large correlations (all exceeding Cohen benchmark for large effects [65]) with established resilience measures are consistent with the WONE Index measuring conceptually related but distinct aspects of stress resilience, as would be expected given its emphasis on specific behavioral resources and demands-resources integration versus the trait-based conceptualizations of the CD-RISC and BRS. These patterns support construct validity while indicating that the WONE Index captures additional variance beyond established measures.

#### Concurrent Validity

The measure demonstrated excellent concurrent validity with theoretically related mental health and well-being outcomes, with all correlations in the expected directions and magnitudes.

##### Stress-Mental Health Relationships

The Stress Subscale showed strong positive correlations with both depressive symptom measures (PHQ-8 and PROMIS-SF-8a) and anxiety symptoms (GAD-7). Conversely, the Stress Subscale demonstrated a strong negative correlation with well-being (WHO-5), indicating that higher stress is associated with lower well-being, as expected.

##### Resilience-Mental Health Relationships

The Resilience Subscale showed strong protective relationships with mental health outcomes. Higher resilience scores were associated with lower depressive symptoms on both measures, lower anxiety symptoms, and higher well-being.

##### Full WONE Index Concurrent Validity

The Full WONE Index demonstrated excellent concurrent validity across all mental health outcomes, with large negative correlations with depressive and anxiety symptoms and a large positive correlation with well-being. These strong relationships across all mental health domains support the Full Index as a comprehensive measure of stress resilience capacity.

##### Comparative Performance

Notably, the WONE Index demonstrated stronger correlations with mental health outcomes than established resilience measures and comparable correlations with the PSS for stress-related outcomes (refer to Table 2).

### Discriminant Validity

The WONE Index showed appropriate discriminant validity when examined against personality traits, with one theoretically meaningful exception that actually supports construct validity.

#### Neuroticism (Supporting Evidence for Construct Validity)

The Full WONE Index demonstrated a strong negative correlation with neuroticism. Rather than representing a discriminant validity concern, this relationship provides important construct validity evidence. Neuroticism, characterized by heightened stress reactivity and emotional instability, would be expected to show a strong negative relationship with stress resilience capacity. This correlation suggests our measure successfully captures individual differences in stress vulnerability and resilience resources, making it particularly valuable for identifying individuals who may benefit most from resilience-building interventions.

#### Other Personality Traits

The Index showed moderate correlations with agreeableness and conscientiousness, suggesting some overlap with these adaptive personality traits, though correlations remained below the threshold indicating construct redundancy. The measure showed a smaller correlation with extraversion and was essentially uncorrelated with openness, demonstrating good discrimination from these personality dimensions.

### Discriminant Validity Between Domains

HTMT ratios were calculated to assess discriminant validity between measurement domains. All HTMT values fell below the conservative threshold of 0.85, with most below 0.70, supporting discriminant validity between domains while confirming their theoretical relationships.

#### Stress Domain Relationships

The 3 stress domains showed moderate to strong relationships, confirming that they capture related but distinct stress experiences (refer to Table S7 in Multimedia Appendix 1).

#### Resilience Domain Relationships

Resilience domains demonstrated appropriate discriminant validity. The strongest relationships were observed between conceptually related domains (Emotion Regulation and Coping ↔ Sleep; Purpose and Prosociality ↔ Social Connection), supporting the theoretical structure while confirming domain distinctiveness (refer to Table S8 in Multimedia Appendix 1).

### Internal Consistency

The CR estimate for the overall scale was 0.84. Estimates for individual factors ranged from 0.62 to 0.96, with 9 of 10 factors exceeding the 0.70 threshold for acceptable reliability. Eight factors achieved good to excellent reliability (CR ≥0.80; refer to Table 3).

**Table 3.** Factor-level test-retest reliability and internal consistency.

Subscale and factor	ICC^a^ (95% CI)	CR^b^	AVE^b^
WONE^c^ Index	0.90 (0.87-0.93)	—	—
Stress subscale	0.84 (0.80-0.88)	—	—
Personal Stress factor	0.90 (0.87-0.92)	0.90	0.64
Work Stress factor	0.89 (0.85-0.91)	0.91	0.67
Burnout factor	0.87 (0.82-0.90)	0.84	0.58
Resilience subscale	0.90 (0.87-0.92)	—	—
Emotion Regulation and Coping factor	0.95 (0.93-0.96)	0.89	0.49
Social Connectedness factor	0.93 (0.91-0.95)	0.94	0.74
Purpose and Prosociality factor	0.93 (0.91-0.95)	0.83	0.49
Sleep factor	0.90 (0.87-0.93)	0.83	0.51
Dietary Intake factor	0.90 (0.86-0.92)	0.62	0.31
Physical Activity factor	0.81 (0.74-0.85)	0.81	0.70
Perseverative Thinking factor	0.79 (0.72-0.84)	0.89	0.80

a ICC: intraclass correlation coefficient.

b Composite reliability (CR) and average variance extracted (AVE) estimates are not applicable to the full WONE Index, as it is calculated as a composite of 2 separate confirmatory factor models, rather than being estimated as a latent factor within a single measurement model.

c WONE: Walking on Earth

The Dietary Intake factor showed marginal reliability (CR=0.62 and AVE=0.31), likely reflecting the conceptual diversity of dietary behaviors assessed (caffeine reliance, processed food consumption, and nutritious diet quality). The marginal reliability of the Dietary Intake factor likely reflects the conceptual diversity of dietary behaviors rather than measurement inadequacy. Despite the lower statistical indicators, these items were retained due to their theoretical importance in the resilience framework and the nascent state of dietary behavior measurement in resilience research.

### Test-Retest Reliability

The WONE Index demonstrated excellent temporal stability across all major scales over 3 weeks (Table 3). The Full WONE Index showed excellent test-retest reliability, indicating highly consistent measurement of overall stress resilience capacity over time. Both subscales also demonstrated strong temporal stability, with the Resilience Subscale exhibiting excellent reliability and the Stress Subscale showing good reliability.

These findings indicate that the WONE Index provides a stable and consistent measurement of both stress and resilience resources across time. The excellent temporal stability of the WONE Index supports its use as a reliable assessment tool for tracking stress resilience capacity. The high test-retest reliability coefficients indicate that observed changes in WONE scores over time reflect true changes in an individual’s stress resilience capacity rather than measurement error, making the instrument suitable for monitoring intervention effects and tracking progress.

Individual WONE factors also showed strong to excellent test-retest reliability across the 3-week interval (Table 3). Nine of 10 factors achieved excellent temporal stability (ICC >0.85).

### Incremental Validity Results

The WONE Index provided significant incremental prediction over a combination of existing gold-standard measures. All hierarchical regression models demonstrated statistically significant incremental validity, indicating that the WONE Index provides meaningful prediction beyond established measures across all mental health outcomes tested. The WONE Index’s superior correlational performance relative to established resilience measures and comparable performance to the PSS provides the foundation for these incremental validity findings.

#### Model 1: Beyond Perceived Stress Scale

The WONE Full Index provided significant incremental prediction beyond the PSS across all outcomes. Even after accounting for perceived stress, the WONE explained additional variance in depressive symptoms (PHQ-8: Δ*R*²=0.10, *P*<.001; PROMIS-SF-8a: Δ*R*²=0.06, *P*<.001; GAD-7: Δ*R*²=0.07, *P*<.001; and WHO-5: Δ*R*²=0.16, *P*<.001).

#### Model 2: Beyond Established Resilience Measures

##### Model 2a (Beyond CD-RISC)

The WONE Index demonstrated substantial incremental validity beyond the CD-RISC across all outcomes, with particularly strong incremental prediction for depressive symptoms and well-being (PHQ-8: Δ*R*²=0.32, *P*<.001; PROMIS-SF-8a: Δ*R*²=0.31, *P*<.001; GAD-7: Δ*R*²=0.31, *P*<.001; and WHO-5: Δ*R*²=0.26, *P*<.001).

##### Model 2b (Beyond BRS)

Similar patterns were observed when testing incremental validity beyond the BRS, with significant ΔR² values across all mental health outcomes (PHQ-8: Δ*R*²=0.31, *P*<.001; PROMIS-SF-8a: Δ*R*²=0.31, *P*<.001; GAD-7: Δ*R*²=0.25, *P*<.001; and WHO-5: Δ*R*²=0.31, *P*<.001).

### Model 3: Beyond Combined Established Measures

Most importantly, the WONE Index provided significant incremental prediction even beyond the combination of both established stress and resilience measures (PSS, CD-RISC, and BRS). This represents the most stringent test of incremental validity, as it demonstrates that WONE adds meaningful prediction beyond current best practices that would use both stress and resilience assessments (PHQ-8: Δ*R*²=0.11, *P*<.001; PROMIS-SF-8a: Δ*R*²=0.07, *P*<.001; GAD-7: Δ*R*²=0.07, *P*<.001; and WHO-5: Δ*R*²=0.11, *P*<.001).

### Measurement Invariance for Stress

#### Measurement Invariance Testing Across Gender

Multigroup CFA indicated good configural fit across women (n=164) and men (n=142), suggesting a consistent factor structure (*χ*²_66_=115.71; *P*<.001; CFI=0.98; TLI=0.96; RMSEA=0.05; and SRMR=0.06). Constraining factor loadings (metric model) maintained a strong fit (*χ*²_76_=125.58; *P*<.001; CFI=0.98; TLI=0.97; RMSEA=0.05; and SRMR=0.06), supporting metric invariance. Adding intercept constraints (scalar model) yielded an acceptable fit (*χ*²_86_=151.50; *P*<.001; CFI=0.97; TLI=0.96; RMSEA=0.05; and SRMR=0.07), supporting latent mean comparisons. The strict model (residual variances constrained) also fit well (*χ*²_99_=163.14; *P*<.001; CFI=0.97; TLI=0.97; RMSEA=0.05; and SRMR=0.07), supporting full invariance across gender.

#### Measurement Invariance Across Race

Participants were grouped as White (n=211) and non-White (n=95). Configural fit was strong (*χ*²_66_=100.62; *P*=.01; CFI=0.99; TLI=0.98; RMSEA=0.04; and SRMR=0.05), indicating consistent structure. The metric model (loadings constrained) also showed excellent fit (*χ*²_76_=100.62; *P*=.03; CFI=0.99; TLI=0.98; RMSEA=0.03; and SRMR=0.05). Scalar constraints (intercepts) retained good fit (*χ*²_86_=111.65; *P*=.03; CFI=0.99; TLI=0.99; RMSEA=0.03; and SRMR=0.04), allowing for latent mean comparisons. The strict model (residuals constrained) showed acceptable fit (*χ*²_99_=129.24; *P*=.02; CFI=0.97; TLI=0.98; RMSEA=0.03; and SRMR=0.045), indicating full invariance across race.

#### Measurement Invariance Across Age Groups

Participants were split into ages 20‐39 years (n=175) and 40‐64 years (n=131). The configural model demonstrated good fit (*χ*²_66_=95.23; *P*=.01; CFI=0.99; TLI=0.98; RMSEA=0.04; and SRMR=0.06). Metric invariance was supported with excellent fit when loadings were constrained (*χ*²_76_=102.98; *P*=.02; CFI=0.99; TLI=0.98; RMSEA=0.03; and SRMR=0.06). The scalar model (adding intercept constraints) showed a strong fit (*χ*²_86_=127.18; *P*=.003; CFI=0.98; TLI=0.98; RMSEA=0.04; and SRMR=0.06), permitting latent mean comparisons. The strict model (residuals constrained) also fit well (*χ*²_99_=143.23; *P*<.002; CFI=0.98; TLI=0.98; RMSEA=0.04; and SRMR=0.06), indicating strict invariance across age groups.

### Measurement Invariance for Resilience Resources

#### Measurement Invariance Across Gender

We evaluated measurement invariance for the Resilience Resources model across gender (women: n=164 and men: n=142) using multigroup CFA. The configural model demonstrated good fit (*χ*²_886_=1223.58; *P*<.001; CFI=0.94; TLI=0.93; RMSEA=0.04; and SRMR=0.08), indicating a consistent factor structure across groups. Constraining factor loadings in the metric model yielded similar fit (χ²_917_=1288.44; *P*<.001; CFI=0.93; TLI=0.92; RMSEA=0.04; and SRMR=0.08), supporting equivalence of loadings. The scalar model, which additionally constrained item intercepts, also showed good fit (*χ*²_949_=1360.84; *P*<.001; CFI=0.92; TLI=0.92; RMSEA=0.04; and SRMR=0.09). The strict model, which further constrained residual variances, continued to meet fit thresholds (*χ*²_988_=1413.10; *P*<.001; CFI=0.92; TLI=0.92; RMSEA=0.04; and SRMR=0.09), supporting full measurement invariance across gender.

#### Measurement Invariance Across Race

We next assessed invariance across race, comparing White (n=211) and non-White (n=95) participants. The configural model demonstrated good fit (*χ*²_886_=1326.95; *P*=.01; CFI=0.92; TLI=0.91; RMSEA=0.04; and SRMR=0.07), indicating a consistent factor structure. The metric model, which constrained factor loadings, showed similar fit (*χ*²_917_=1363.49; *P*<.001; CFI=0.92; TLI=0.91; RMSEA=0.04; SRMR=0.07). The scalar model, which additionally constrained item intercepts, maintained good fit (*χ*²_949_=1433.49; *P*<.001; CFI=0.91; TLI=0.91; RMSEA=0.04; and SRMR=0.07). The strict model, which further constrained residual variances, also met fit thresholds (*χ*²_988_=1540.42; *P*<.001; CFI=0.90; TLI=0.90; RMSEA=0.04; and SRMR=0.07), supporting full measurement invariance across race.

#### Measurement Invariance Across Age Groups

Participants were grouped by age 20‐39 years (n=175) and 40‐64 years (n=131). The configural model demonstrated acceptable fit (*χ*²_886_=1352.55; *P*<.001; CFI=0.92; TLI=0.91; RMSEA=0.04; and SRMR=0.07). Metric invariance was supported with excellent fit when loadings were constrained (*χ*²_917_=1385.98; *P*<.001; CFI=0.92; TLI=0.91; RMSEA=0.04; and SRMR=0.08). The scalar model (adding intercept constraints) showed strong fit (*χ*²_949_=1458.55; *P*<.001; CFI=0.91; TLI=0.90; RMSEA=0.04; and SRMR=0.08), permitting latent mean comparisons. The strict model (residuals constrained) also fit well (*χ*²_988_=1542.30; *P*<.001; CFI=0.90; TLI=0.90; RMSEA=0.04; and SRMR=0.08), indicating strict invariance across age groups.

### Weighting

#### Empirical Weight Distribution

The regression-derived empirical weights revealed a clear hierarchical structure dominated by psychological and stress-related constructs (Table 4). Emotion Regulation and Coping emerged as the most critical predictor (44.6%), followed by Personal Stress (16.3%) and Perseverative Cognition (8.3%). Notably, Physical Activity received minimal empirical weighting (0.2%), likely reflecting mediation through other measured constructs rather than a lack of theoretical importance.

**Table 4.** Empirical, theoretical, and final hybrid weights.

Construct	Empirical weight, %	Theoretical weight, %	Hybrid weight, %	Final weight, %
Emotion Regulation and Coping	44.6	20.0	32.3	31
Personal Stress	16.3	10.0	13.2	13
Social Connectedness	7.8	18.0	12.9	12
Perseverative Thinking	8.3	14.0	11.2	11
Sleep	6.9	9.0	7.9	8
Dietary Intake	6.2	5.0	5.6	5
Burnout	5.3	5.0	5.2	5
Physical Activity	0.2	7.0	3.6	5
Work Stress	1.0	7.0	4.0	5
Purpose and Prosociality	3.9	5.0	4.5	5

#### Theoretical Weight Rationale

Theoretical weights addressed empirical limitations while ensuring comprehensive coverage of modifiable resilience factors. Social Connectedness received high theoretical weighting based on robust meta-analytic evidence linking social support to stress resilience outcomes [66,67]. Sleep and Physical Activity received substantial theoretical weights given their well-documented roles in stress buffering and physiological recovery mechanisms [68-70].

Work Stress received meaningful theoretical weighting despite minimal empirical contribution, reflecting complex nonlinear relationships observed in organizational research, where higher work demands correlate positively with resilience in certain populations [71], suggesting stress inoculation effects [72] or self-selection mechanisms.

#### Hybrid Weight Validation

Cross-sectional validation demonstrated superior performance of the hybrid weighting approach across all criterion measures (Table 5). The weighted approach showed consistent improvements over unweighted alternatives, with particularly strong gains for primary resilience measures: CD-RISC (+0.06 vs unweighted) and BRS (+0.08 vs unweighted).

**Table 5.** Validation performance comparison.

Outcome measure	Hybrid weighted	Unweighted	Item-level	Best performing version
CD-RISC^a^	0.75^b^	0.68^b^	0.73^b^	Weighted
BRS^c^	0.74^b^	0.67^b^	0.71^b^	Weighted
PSS-4^d^	−0.81^b^	−0.77^b^	−0.79^b^	Weighted
PHQ-8^e^	−0.77^b^	−0.76^b^	−0.77^b^	Item-level
PROMIS-SF-8a^f^	−0.79^b^	−0.77^b^	−0.79^b^	Weighted
GAD-7^g^	−0.77^b^	−0.74^b^	−0.73^b^	Weighted
WHO-5^h^	0.83^b^	0.81^b^	0.84^b^	Item-level

a CD-RISC: Connor-Davidson Resilience Scale.

b *P*<.001

c BRS: Brief Resilience Scale.

d PSS-4: Perceived Stress Scale-4.

e PHQ-8: Patient Health Questionnaire-8.

f PROMIS-SF-8a: Patient-Reported Outcomes Measurement Information System Short Form 8a.

g GAD-7: Generalized Anxiety Disorder-7.

h WHO-5: World Health Organization-5 Well-Being Index.

#### Validation Performance Summary

The hybrid approach demonstrated meaningful improvements across resilience-related outcomes while maintaining strong correlations with mental health indicators. The greatest improvements occurred for measures most directly assessing resilience capacity (CD-RISC and BRS), suggesting the theoretical components successfully enhanced the measurement of core resilience constructs.

Notably, the hybrid approach outperformed both purely unweighted and item-level averaging approaches, indicating that the systematic integration of empirical prediction with theoretical importance creates optimal measurement properties. The modest improvement for depression (PHQ-8) likely reflects the already strong empirical weighting of constructs most relevant to depressive symptoms.

#### Final Weight Structure and Clinical Implications

The final hybrid weights create a theoretically coherent hierarchy that preserves predictive validity while ensuring comprehensive intervention guidance. Emotion Regulation and Coping maintains prominence, consistent with its central role in stress resilience frameworks. Core stress and cognitive constructs receive substantial representation: Personal Stress, Social Connectedness, and Perseverative Thinking.

Critically, all behavioral and contextual factors achieve meaningful weighting for intervention purposes: Sleep, Physical Activity, Work Stress, Dietary Intake, Burnout, and Compassion/Gratitude/Meaning. This distribution supports the WONE Index’s dual function as both a predictive assessment and behavioral intervention guidance system.

Longitudinal validation using Time 1 WONE scores to predict Time 2 outcomes (n=203) further confirmed the superior predictive performance of the weighted approach across all validation measures, though detailed longitudinal analyses are beyond the scope of the current phase.

## Discussion

### Principal Findings

The WONE Index was developed and validated through a 2-phase process to progressively refine its measurement model. Phase 1 provided strong initial psychometric evidence, establishing a 6-factor structure organized into 2 higher-order domains: Stress Load (Work Stress, Personal Stress, and Burnout) and Resilience Resources (Resilience Skills and Beliefs, Social Support, and Sleep). While statistically robust, this model did not fully capture the multidimensional and dynamic nature of resilience, particularly with respect to health behaviors and cognitive processes.

Phase 2 expanded and refined this framework into a 10-factor structure organized within 2 higher-order domains, which we propose as the finalized structure. Seven resource-related factors (Emotion Regulation and Coping, Social Connection, Purpose and Prosociality, Sleep, Physical Activity, Dietary Intake, and Perseverative Thinking) and 3 stress factors (Work Stress, Personal Stress, and Burnout) were identified and grouped into the higher-order domains of Stress Load and Resilience Resources. In both phases, we followed standard CFA practice by incorporating theoretically justified modifications suggested by MIs, including correlated residuals within conceptually related item sets. These refinements improved model fit while preserving the theoretical structure. Model fit indices for both Phase 1 and Phase 2 were strong, but the Phase 2 model offered comparable psychometric performance while adding domains that improved interpretability and intervention relevance, making it the preferred structure. By incorporating additional factors, the final model allows for greater precision in identifying areas of vulnerability and creates more actionable pathways for intervention.

Importantly, the Index allows us to move beyond identifying whether someone is “resilient” to understanding how their system is functioning. It captures systemic imbalances in how individuals perceive, feel, adapt, and respond to stress, thereby holistically measuring resilience and highlighting where interventions should target. For example, someone experiencing high stress load but with depleted resources may benefit most from micro-moment stress-reduction strategies that support daily functioning before resource-building can take hold. Conversely, individuals with low external stressors but insufficient resilience resources may require preventive interventions to expand their repertoire of coping and regulatory skills.

This perspective also acknowledges paradoxical cases such as “skin-deep resilience,” in which individuals may appear psychologically resilient to chronic stress, yet their bodies still carry physiological costs (eg, accelerated aging, immune dysregulation, and cardiovascular risk). In such cases, building resilience resources remains essential, as resource strengthening may buffer against hidden biological wear-and-tear even when stress load is not consciously perceived as high [73,74]. By integrating both stress load and resilience resources, the WONE Index provides a nuanced lens for identifying vulnerability, tailoring interventions, and supporting resilience development across populations and contexts.

The WONE Index also demonstrated excellent temporal stability, strong reliability across scales, and robust validity with multiple established measures of resilience, stress, and mental health outcomes. Together, this evidence supports the Index as both a rigorous scientific tool and a practical framework for understanding resilience in diverse populations.

The hybrid weighting methodology is a key innovation of this work. It integrates empirical weights derived from predictive modeling of mental health and well-being outcomes with theoretical weights grounded in resilience science and dynamical systems constructs (eg, Sleep, Social Connection, and Emotion Regulation). This dual approach produces scores that are both predictively robust and have practical utility in digital health and applied psychology.

Conceptually, this framework aligns with multidimensional outcome systems such as the Treatment Outcome Package [75], which emphasizes comprehensive assessment across multiple domains to guide individualized feedback and treatment. The WONE Index adopts a similar logic by balancing empirical evidence with theoretical modifiability, ensuring that domain scores capture both predictive value and potential for change through intervention. This approach enhances the Index’s relevance for digital health applications by identifying high-impact targets while also laying the foundation for future integration of predictive modeling approaches to further enhance precision and personalization. In doing so, it bridges traditional clinical feedback models with data-driven precision frameworks, offering a scalable approach to personalized resilience assessment.

Together, these findings establish the WONE Index as a comprehensive, multidimensional, and psychometrically rigorous measure of resilience, conceptualized as adaptive capacity emerging from the balance between stress load and resilience resources, and designed to both measure resilience and inform targeted intervention.

### Comparison With Previous Work

O’Donohue et al [76] identified 45 distinct measurement approaches with substantial heterogeneity in stress resilience conceptualization and operationalization. Critical limitations documented across existing measures include: (1) most measures assess stress or resilience separately rather than their dynamic interaction, with only 17.5% using resilience measures and many relying solely on stress indices; (2) predominant use of trait-based rather than process-based conceptualizations (eg, CD-RISC, BRS, and RSA which focus on stable characteristics), limiting sensitivity to temporal change; and (3) limited validation for repeated measurement contexts, with most instruments designed for single-timepoint assessment [8]. In addition, widely used resilience measures—including the CD-RISC, BRS, and RSA—focus predominantly on perceptual attitudes (“I can deal with whatever comes”) rather than behaviorally specific, modifiable resources, and do not integrate health behavior determinants—including sleep, physical activity, and nutrition—documented as protective factors that buffer stress and promote resilience [20,21,77-80].

The WONE Index addresses these gaps through 3 key distinctions. First, it integrates stress load and resilience resources within a unified framework, operationalizing the demands-resources balance emphasized by JD-R theory rather than measuring constructs separately [12]. Second, it provides comprehensive domain coverage spanning psychological resources (emotion regulation, coping, and perseverative cognition), social resources (connection and support), meaning-oriented resources (purpose, gratitude, and prosociality), and health behavioral resources (sleep, physical activity, and nutrition)—a combination rarely found in existing validated measures to our knowledge. Third, it emphasizes behaviorally specific, modifiable capacities providing concrete intervention targets for digital health applications. However, longitudinal validation is needed to establish sensitivity to intervention-related change, and comparative effectiveness research must determine whether this integrated approach provides advantages over using established measures in combination, while independent replication by researchers without commercial affiliations is essential for confirming generalizability across diverse populations and contexts.

The WONE Index contributes to a growing literature emphasizing that stress and resilience are not opposite poles of a single construct but distinct domains with unique predictive value. Similar to existing resilience frameworks [4,11], our results underscore the centrality of regulatory skills, social connection, emotion, and meaning-making, but the Index also extends previous tools in several important ways. Unlike widely used measures that treat demands and resources as isolated constructs (eg, PSS [18], CD-RISC [20], and BRS [21]), the Index embeds them in a unified structure reflecting how these factors interact as interdependent subsystems. This operationalization enables the measure to serve dual purposes: scientifically, it captures multidomain adaptive capacity aligned with JD-R and systems-based models; practically, it functions as an intervention guidance system that balances measurement rigor with intervention utility.

The 10-factor structure within 2 higher-order domains provides a multidimensional systems approach to resilience that begins to approximate a network of interdependent resilience domains by capturing distinct contributions from cognitive, behavioral, social, and emotional domains, each of which showed robust factor loadings and validity evidence in Phase 2. Cognitive (eg, perseverative thinking), social (social connection and purpose/prosociality), emotion (eg, emotion regulation and positive affect), health behavior (eg, sleep, physical activity, and diet), and stress exposures (eg, personal, work, and burnout) are all included, creating a systemic framework rather than a 1D scale, which has been popular in established measures of resilience.

This expanded domain coverage forms the foundation of its systems perspective. This aligns with allostatic models, JD-R, and complex dynamical systems models of psychopathology, which emphasize how individuals may become “stuck” in maladaptive states or transition toward adaptive states depending on system-level dynamics [81]. By conceptualizing resilience as adaptive capacity emerging from the balance between stress load and resilience resources, the WONE Index provides a practical operationalization of these theoretical models.

As mentioned in the previous paragraph, the WONE index builds directly on the JD-R framework [12,13], which posits that health, well-being, and performance are determined by the interplay between demands (eg, workload, role conflict, and emotional strain) and resources (eg, social support, coping strategies, and recovery experiences). Our higher-order domains map closely to this model: Stress Load reflects job and life demands, while Resilience Resources reflect the protective assets available to meet them. Importantly, the JD-R framework distinguishes acute strain driven by demands from the chronic exhaustion that develops into burnout. The WONE Index mirrors this theoretical distinction by separately modeling work stress and burnout, offering a psychometric tool for examining how short-term strain may evolve into longer-term depletion and maladaptive system states.

Beyond alignment, the Index extends JD-R in 2 important ways. First, it broadens the scope by incorporating personal, societal, and health behavior domains, increasing relevance across multiple life contexts rather than being limited to occupational settings. Second, the hybrid weighting methodology adds precision to JD-R by quantifying the relative impact of different resources. Whereas JD-R theory broadly emphasizes the buffering role of resources, the WONE Index specifies which domains—such as sleep, coping, or social connection—are both theoretically central and empirically predictive of outcomes such as depression, anxiety, and well-being. In this way, the Index operationalizes the JD-R concept of resources while also extending it to a wider range of life demands and contexts.

### Applications to Digital Mental Health

The WONE Index advances digital mental health measurement in several important ways.

First, it was developed and validated specifically within digital delivery contexts, ensuring its psychometric properties hold when administered via web and mobile interfaces rather than assuming transferability from traditional formats. The index was validated via a digital survey/platform, providing confidence that observed reliability and validity reflect real-world digital administration rather than performance under idealized controlled conditions.

Second, the Index enables a measurement-based care approach at scale. Traditional assessment typically occurs at discrete time points (eg, intake and discharge) with results informing clinician-delivered interventions. In contrast, the WONE Index supports continuous assessment cycles where monthly administration generates updated resilience profiles that automatically inform algorithmic personalization of intervention content. This creates feedback loops between assessment and intervention that are difficult to achieve in traditional service delivery models, allowing platforms to adaptively adjust recommendations as users’ stress-resource balance shifts over time.

Third, the multidimensional structure provides actionable granularity for automated intervention matching. Rather than producing a single resilience score requiring clinician interpretation to determine intervention targets, the 10 factors within 2 overarching domains assessed in the WONE Index directly map onto intervention modules within the platform. Low scores on emotion regulation trigger recommendations for regulation-focused techniques, depleted social connection scores prompt social relationship-focused interventions, and poor sleep activates sleep hygiene interventions. This direct assessment-to-intervention mapping enables digital platforms to deliver truly personalized care pathways without requiring human clinical judgment for every user.

Fourth, the measure’s design supports population-level insights alongside individual assessment. Aggregated anonymized data reveal organizational stress patterns, high-risk subgroups, and systemic factors affecting workforce resilience. This dual-level utility, individual personalization plus population surveillance, distinguishes the Index from traditional measures designed solely for individual clinical assessment. Organizations can identify when specific teams show declining resilience resources or elevated stress load, enabling proactive organizational interventions before individual crises emerge.

Finally, the hybrid weighting methodology reflects digital health’s unique requirements. Pure empirical weighting maximizes prediction but may undervalue behaviorally modifiable domains showing weaker cross-sectional associations yet greater intervention responsiveness. Theoretical weighting ensures the Index prioritizes domains where digital platforms can deliver effective interventions (eg, sleep, physical activity, and social connection) even when these show modest concurrent prediction. This methodology acknowledges that digital health assessment serves intervention guidance, not just prediction, requiring weights that balance predictive validity with behavioral actionability.

Together, these features distinguish the WONE Index from existing stress and resilience measures, which typically assess either protective or risk factors in isolation, rely on static trait-based designs, and lack validation for digital health or workplace contexts. By integrating stress load and resilience resources within a unified, multidomain structure specifically designed for digital mental health contexts and linking assessment directly to intervention personalization, the WONE Index operationalizes the demands-resources framework for temporal tracking through repeated administration in applied digital settings.

### Strengths

This study has several notable strengths. First, it used a multiphase design, beginning with exploratory development and culminating in confirmatory validation. This sequential approach provides both a rigorous psychometric foundation and evidence of generalizability. Second, the WONE Index advances the field by integrating multiple domains of resilience into a hierarchical structure, capturing stress load alongside resilience resources. This systemic framing reflects how resilience operates across cognitive, social, behavioral, and meaning-oriented processes rather than as a single dimension.

Third, the Index demonstrated robust psychometric qualities, including strong reliability, validity across multiple criteria, and temporal stability, supporting its use for both research and applied purposes. Fourth, the hybrid weighting methodology represents a novel contribution to measuring development, balancing empirical prediction with theoretical modifiability and enhancing the practical utility of final scores.

### Limitations

Several limitations should also be noted. First, the data were cross-sectional, which precludes causal inference. While test-retest analyses support temporal stability, the design limits the ability to determine whether changes in stress load or resilience resources precede improvements in outcomes. Future longitudinal studies are needed to examine how resilience processes evolve over time and in response to intervention.

Second, the study relied on self-report measures, which introduces potential for bias, such as recall inaccuracy or social desirability effects. We sought to mitigate these issues by using validated instruments with well-established psychometric properties and by using a brief, single-session survey format to minimize fatigue-related error. Nevertheless, self-report bias could have influenced observed relationships among constructs. Future studies should integrate behavioral and physiological indicators (eg, ecological momentary assessment, wearables, and biomarkers) to strengthen validity and reduce reliance on self-report alone.

Third, although the sample was diverse and measurement invariance was supported across gender, race, and age, the use of online convenience sampling (CloudResearch and Prolific) may limit generalizability to digitally-engaged populations. Participants were primarily from the United States and the United Kingdom and were predominantly White, which may constrain cultural generalizability. Accordingly, future research should aim to validate the WONE Index across more diverse cultural, linguistic, and global populations to ensure measurement equivalence and capture potential cultural differences in how stress load and resilience resources manifest.

Fourth, brief item pools for some domains such as diet and physical activity, while necessary for reducing participant burden, may have limited construct breadth. The Dietary Intake factor, in particular, showed lower reliability, representing a known measurement limitation. We retained this domain due to its theoretical relevance and behavioral actionability in digital health contexts.

In addition, these health behavior domains may capture variance from aspects of resilience not fully represented in other adaptive resource domains (eg, energy balance or embodied forms of adaptation). It is also possible that shared variance among closely related constructs may have partially attenuated their distinct contributions. Future research should be mindful that diet and physical activity may relate more strongly to multimodal and objective data sources (eg, wearables and ecological monitoring) given their greater overlap with physiological processes compared to psychosocial resources.

Further, while the hybrid weighting system enhances both predictive power and practical relevance, the process of assigning theoretical weights remains partly subjective. We sought to balance theoretical rationale with empirical model fit, but weighting decisions could still introduce bias in the relative importance of certain domains. Future work should refine these weightings through stakeholder engagement and cross-validation in applied contexts.

Finally, the WONE Index was developed within a digital health implementation context, providing access to large-scale user samples and enabling iterative refinement based on real-world engagement. While this design addresses documented measurement gaps through integrated assessment of stress and resilience resources, establishing whether this approach provides practical advantages over existing measures requires additional validation. Future research should examine predictive validity for clinical outcomes and intervention response, longitudinal sensitivity to change in intervention contexts, and generalizability across demographic and cultural groups.

Comparative effectiveness research will be valuable to determine whether integrated assessment offers advantages over domain-specific measures used in combination, or whether different measurement approaches serve complementary purposes depending on the implementation context. Independent validation by researchers without commercial affiliations will also be valuable for confirming generalizability and providing an unbiased evaluation of the measure’s comparative utility across different use cases.

### Future Directions

This work lays the foundation for several important lines of future research. First, longitudinal studies are needed to examine how resilience resources and stress load interact over time and to test whether the WONE Index is sensitive to intervention-related changes. Such research will help clarify the dynamic nature of adaptive capacity and resilience plasticity and help examine whether certain domains play different roles in resilience decline versus recovery. This work may also reveal that the factors contributing to stress-related problems differ from those most critical for recovery, information that could further refine how digital platforms prioritize and sequence intervention recommendations to maximize impact during different phases of the resilience process.

Second, clinical applications represent a critical next step. Embedding the WONE Index into treatment contexts could provide insights into how resilience factors contribute to therapeutic outcomes, as well as identify which domains serve as leverage points for recovery. The hierarchical, multidomain structure is particularly well-suited for monitoring differential change across domains during intervention.

Third, contextual and multimethod integration holds promise for advancing ecological validity. Pairing the Index with ecological momentary assessment could capture the contexts in which stress triggers arise and the adaptive strategies mobilized in response, illuminating how resilience unfolds in daily life. This could be further enhanced through pairing with objective markers, including wearable-derived measures (eg, sleep patterns, activity, and heart rate variability) and biomarkers (eg, cortisol and inflammatory cytokines). Such multimethod approaches would reduce reliance on self-report alone, allow researchers to examine how subjective and biological processes align or diverge, and provide a more complete picture of adaptive capacity as a multilevel system.

Fourth, cross-cultural research is needed to explore how resilience processes manifest across diverse sociocultural contexts. While the Index demonstrated measurement invariance across gender, race, and age, expanding to other cultural and occupational groups would strengthen its generalizability and highlight context-specific resilience mechanisms.

Finally, continued methodological innovation will be essential for advancing resilience science. While the hybrid weighting system provides a balanced approach to scoring, the WONE Index also creates opportunities for more advanced analytic methods that can further expand resilience science. Machine learning can extend beyond traditional statistical models by prioritizing prediction and generalizability. It can leverage the Index’s structure to detect complex nonlinear interactions, uncover latent resilience profiles through clustering methods, and integrate multimodal data (eg, self-report, wearables, and biomarkers). Machine learning’s emphasis on out-of-sample accuracy and scalability makes it particularly useful for precision prediction in digital health contexts.

Future research can also incorporate Bayesian and systems-based approaches to deepen mechanistic understanding. Bayesian approaches can model uncertainty around resilience estimates, incorporate previous theoretical knowledge, and improve estimation in smaller or intensive longitudinal samples such as ecological momentary assessment. Additional frameworks, including network analysis, dynamical systems modeling, and mixture modeling, could further illuminate how resilience operates as a system, identify leverage points, and detect early warning signals of maladaptive change. Together, these approaches position the WONE Index as both a measurement tool and a platform for advancing predictive and mechanistic models of resilience.

### Conclusion

The WONE Index successfully bridges scientific rigor and practical utility, addressing the assessment-intervention gap that has limited resilience research impact in applied settings. Strong incremental validity beyond gold-standard measures demonstrates that the Index captures unique aspects of stress-resilience capacity not assessed by existing tools. The methodological innovation of hybrid weighting—balancing empirical prediction with theoretical modifiability—establishes a strengthened standard for intervention-focused measurement development.

By simultaneously assessing stress load and resilience resources within a unified framework specifically designed for digital delivery, the Index enables personalized intervention matching at scale while maintaining rigorous psychometric standards. As digital mental health expands, measures that satisfy both scientific and practical requirements will be essential for enhancing treatment effectiveness and accessibility. The WONE Index provides a scientifically grounded foundation for this evolution, serving as both a research tool and a platform-integrated assessment system.

## Supplementary material

10.2196/81714 Multimedia Appendix 1 Power analyses for Phase 1 (N=1005) and Phase 2 (N=306), Phase 1 and Phase 2 participant demographics (Tables S1-S2), Phase 1 CFA factor loadings and communalities (Table S3), Phase 1 internal consistency statistics (Table S4), Phase 1 HTMT discriminant validity matrix (Table S5), correlations with established measures (Table S6), comparative fit index (CFA) model modifications, and **Phase 2** heterotrait-monotrait (HTMT) discriminant validity results (Tables S7-S8).

## Abbreviations

AVE: average variance extracted
BFI-10: Big Five Inventory-10
BRS: Brief Resilience Scale
CD-RISC: Connor-Davidson Resilience Scale
CES-D: Center for Epidemiologic Studies Depression
CFA: confirmatory factor analysis
CFI: comparative fit index
CR: composite reliability
*DSM*: *Diagnostic and Statistical Manual of Mental Disorders*
EFA: exploratory factor analysis
GAD-7: Generalized Anxiety Disorder-7
HTMT: heterotrait-monotrait
ICC: intraclass correlation coefficient
IRB: Institutional Review Board
JD-R: job demands-resources
KMO: Kaiser-Meyer-Olkin
MI: modification index
PHQ: Patient Health Questionnaire
PROMIS-SF-8a: Patient-Reported Outcomes Measurement Information System Short Form 8a
PSS: Perceived Stress Scale
RMSEA: root mean square error of approximation
SRMR: standardized root mean square residual
TLI: Tucker-Lewis index
WHO-5: World Health Organization-5 Well-Being Index
WONE: Walking on Earth
ΔR²: R² change
