# Supplementary material for: The WONE Index as a Multidimensional Assessment of Stress Resilience: A Development and Validation Study
Source: J Med Internet Res. 2026 Jan 5;28:e81714. doi: 10.2196/81714 (PMC12768397; doi:10.2196/81714)
Supplement: Multimedia Appendix 1 [file jmir-v28-e81714-s001.pdf]

Multimedia Appendix 1 – Supplementary Materials for:

The WONE Index as a Multidimensional Assessment of Stress Resilience: A Development and Validation Study

Lydia Genevieve Roos, PhD[1]; Destiny Gilliland, BA[1,2]; Kelsey Julian, MA[1,3]; Reeva Misra, MA[1]

[1]Walking on Earth, Ltd, London, United Kingdom

[2]Department of Biobehavioral Health, College of Human Health and Development, The Pennsylvania State University, University Park, PA, United States

[3]Health Psychology PhD Program, College of Humanities and Earth and Social Sciences, University of North Carolina at Charlotte, Charlotte, NC, United States

Corresponding Author:
Lydia Genevieve Roos, PhD
Email: lydia@walkingonearth.com

Contents:

1. Phase 1 Power Analyses
2. Phase 2 Power Analyses
3. Table S1. Phase 1 demographics
4. Table S2. Phase 2 demographics
5. Table S3. Standardized factor loadings and communalities for Phase 1 CFA model
6. Table S4. Phase 1 internal consistency statistics
7. Table S5. Phase 1 HTMT matrix
8. Table S6. Phase 1 correlations between WONE Index and established measures
9. Table S7. HTMT results - Stress subscale
10. Table S8. HTMT results - Resilience Resources subscale
11. CFA Model Modifications
12. References

Phase 1 Power Analyses

EFA and CFA Power Analysis

For both EFA and CFA, we applied MacCallum et al.'s [1] approach based on RMSEA (Root Mean Square Error of Approximation) to determine the required sample size. For EFA, we anticipated testing a model with approximately 25-30 items loading onto 5-7 factors. We conservatively estimated degrees of freedom (df)=200, tested RMSEA of 0.05 (close fit) versus 0.08 (mediocre fit), and N=1000, provided 80% power to detect factor loadings as small as 0.175—well below our minimum threshold of 0.30, yielding >99% power for meaningful loadings. For CFA with estimated df=302, N=1000 provided 80% power to detect RMSEA differences as small as 0.01, ensuring sensitivity to identify even minor model misspecifications when evaluating fit indices (CFI, TLI, RMSEA, and SRMR) against established cutoffs [2].

Internal Consistency Reliability Power Analysis

We used Bonett's method [3] to set our minimum acceptable reliability at 0.70 for subscales and 0.90 for total scales, following standard psychometric guidelines. The standard error of alpha was calculated using: $SE(\alpha)=\sqrt{[(2 * (1-\alpha)^2 * (1+(k-1)*\alpha)^2) / ((k-1) * (n-2))]}$, which showed that 120 participants would provide a 95% confidence interval with a width of ± 0.05 around α=.80. With N=1000, we would have extremely precise reliability estimates, allowing for confident assessment of both overall scale and subscale reliability.

Convergent, Concurrent, and Discriminant Validity Power Analysis

For establishing convergent and concurrent validity through correlation analyses with validated measures, we used $r=\sqrt{[(z\alpha + z\beta)^2 / (n-3)]}$, where $z\alpha=1.96$ ($\alpha=.05$) and $z\beta=0.84$ (power=0.80). With N=1000, we could detect correlations as small as 0.088 with 80% power. For moderate correlations (r>0.30) and large correlations (r>0.50), which we expected between our measure and established convergent measures, our power would exceed 99%. This sensitivity also extends to discriminant validity analyses, ensuring we could confidently detect both the presence of expected relationships and the absence of theoretically inappropriate correlations between the WONE Index and unrelated constructs.

Phase 2 Power Analyses

Confirmatory Factor Analysis Power Analysis

For the CFA, we utilized the MacCallum et al [1] approach. With our planned sample size of 300, our analysis showed that we would be able to detect RMSEA differences as small as 0.02 with 80% power, providing adequate sensitivity for model evaluation within each domain.

Test-Retest Reliability Power Analysis

We applied Zou's [4] method for ICC sample size calculation. For detecting ICC≥0.70 with 80% power, we required 30 participants at Time 2. With anticipated 40-60% retention from 300 participants at Time 1, we

expected 120-180 participants at Time 2, exceeding minimum requirements for establishing test-retest reliability.

Validity Correlations Power Analysis

Using standard power calculations for bivariate correlations, we applied the formula: $r=\sqrt{[(z\alpha + z\beta)^2 / (n-3)]}$, where $z\alpha=1.96$ and $z\beta=0.84$. With N=300, we determined we would be able to detect correlations as small as 0.161 with 80% power. For moderate correlations ($r>0.30$) expected with established measures, our power would exceed 95%.

Incremental Validity Power Analysis

For hierarchical regression analyses, we estimated the minimum detectable effect using: $\Delta R^2 min=8/n$. For our planned sample of N=300, this translated to a minimum detectable R^2 change of 0.03 with 80% power, providing good sensitivity for detecting meaningful incremental prediction (small R^2 changes [$\Delta R^2 > 0.02$]: >90% power; medium R^2 changes [$\Delta R^2 > 0.13$]: >99% power). Together, these results suggest that the study was well powered to detect effects across all planned analysis.

Table S1. Phase 1 demographics (N=1005).

Characteristic	Participants
**Country**	
**USA (n=502)**	
Age (years), mean (SD)	37.2 (9.6)
**Age (years), n (%)**	
18-29 years	122 (24.4)
30-39 years	194 (38.8)
40-49 years	124 (24.8)
50-64 years	60 (12.0)
**UK (n=503)**	
Age (years), mean (SD)	36.0 (10.4)
**Age (years), n (%)**	
18-29 years	155 (31.1)
30-39 years	185 (37.1)
40-49 years	83 (16.7)

			50-64 years	75 (15.1)
	**USA (n=502)**			
		**Gender, n (%)**		
			Woman (cisgender)	254 (50.7)
			Man (cisgender)	237 (47.3)
			Genderqueer or non-conforming	7 (1.4)
			Trans woman	0 (0.0)
			Trans man	1 (0.2)
			Gender not listed or prefer not to answer	2 (0.4)
	**UK (n=503)**			
		**Gender, n (%)**		
			Woman (cisgender)	243 (48.3)
			Man (cisgender)	248 (49.3)
			Genderqueer or non-conforming	1 (0.2)
			Trans woman	1 (0.2)
			Trans man	1 (0.2)
			Gender not listed or prefer not to answer	9 (1.8)
	**USA, (n=502)**			
		**Race and Ethnicity, n (%)**		
			African American / Black	86 (17.1)
			Hispanic/Latinx Ethnicity	3 (3.5)
			East Asian	38 (7.6)
			Hispanic/Latinx Ethnicity	2 (5.3)
			Middle Eastern	4 (0.8)
			Hispanic/Latinx Ethnicity	0 (0)

Native American	4 (0.8)
Hispanic/Latinx Ethnicity	0 (0)
Pacific Islander	3 (0.6)
Hispanic/Latinx Ethnicity	0 (0)
South Asian	6 (1.2)
Hispanic/Latinx Ethnicity	0 (0)
Southeast Asian	16 (3.2)
Hispanic/Latinx Ethnicity	0 (0)
White	358 (71.3)
Hispanic/Latinx Ethnicity	14 (3.9)
**UK (n=503)**	
**Race and Ethnicity, n (%)**	
African American / Black	104 (20.7)
Hispanic/Latinx Ethnicity	0 (0)
East Asian	7 (1.4)
Hispanic/Latinx Ethnicity	1 (14.3)
Middle Eastern	2 (0.4)
Hispanic/Latinx Ethnicity	0 (0)
Native American	2 (0.4)
Hispanic/Latinx Ethnicity	0 (0)
Pacific Islander	2 (0.4)
Hispanic/Latinx Ethnicity	0 (0)
South Asian	16 (3.2)
Hispanic/Latinx Ethnicity	0 (0)
Southeast Asian	8 (1.6)
Hispanic/Latinx Ethnicity	0 (0)

			White		352 (70.0)
				Hispanic/Latinx Ethnicity	1 (0.3)

Table S2. Phase 2 demographics (N=306).

**Characteristic**			**Participants**
**Age (years), mean (SD)**			39.1 (9.6)
**Age (years), n (%)**			
	20-29 years		51 (16.7)
	30-39 years		124 (40.5)
	40-49 years		81 (26.5)
	50-63 years		50 (16.3)
**Gender, n (%)**			
	Woman (cisgender)		161 (52.6)
	Man (cisgender)		140 (45.8)
	Genderqueer or non-conforming		1 (0.3)
	Trans woman		0 (0.0)
	Trans man		1 (0.3)
	Gender not listed or prefer not to answer		0 (0.0)
**Race and Ethnicity, n (%)**			
	African American / Black		47 (15.4)
		Hispanic/Latinx Ethnicity	1 (0.2)
	East Asian		19 (6.2)
		Hispanic/Latinx Ethnicity	0 (0)
	Middle Eastern		4 (1.3)
		Hispanic/Latinx Ethnicity	1 (0.2)
	Native American		2 (0.7)
		Hispanic/Latinx Ethnicity	0 (0)

	Pacific Islander		3 (1.0)
		Hispanic/Latinx Ethnicity	0 (0)
	South Asian		4 (1.3)
		Hispanic/Latinx Ethnicity	0 (0)
	Southeast Asian		7 (2.3)
		Hispanic/Latinx Ethnicity	0 (0)
	White		209 (68.3)
		Hispanic/Latinx Ethnicity	10 (2.9)

Table S3. Standardized factor loadings and communalities for Phase 1 CFA model.

**Construct**	**Factor**	**Domain**	**Std. Loading**	**SE**	**Communality (R^2)**
Cynicism	Burnout	Stress	0.77	0.02	0.59
Disengagement	Burnout	Stress	0.88	0.01	0.77
Lack of Productivity	Burnout	Stress	0.67	0.02	0.45
Anxious at home/ in your personal life	Personal Stress	Stress	0.81	0.02	0.66
Overwhelmed at home/ in your personal life	Personal Stress	Stress	0.81	0.02	0.66
Stressed at home/ in your personal life	Personal Stress	Stress	0.83	0.01	0.69
Sleep Duration	Sleep	Stress	0.52	0.04	0.27
Anxious at work	Work Stress	Stress	0.78	0.02	0.61
Mental Exhaustion	Work Stress	Stress	0.77	0.02	0.59
Overwhelmed at work	Work Stress	Stress	0.74	0.02	0.55
Stressed at work	Work Stress	Stress	0.82	0.01	0.67
Ability to Bounce Back	Resilience Skills and Beliefs	Resilience Resources	0.64	0.02	0.41
Coping Ability	Resilience Skills and Beliefs	Resilience Resources	0.74	0.02	0.55
Effective Coping	Resilience Skills and Beliefs	Resilience Resources	0.64	0.02	0.41

Emotion Regulation	Resilience Skills and Beliefs	Resilience Resources	0.67	0.02	0.45
Growth Mindset	Resilience Skills and Beliefs	Resilience Resources	0.51	0.03	0.27
Meaning and Purpose	Resilience Skills and Beliefs	Resilience Resources	0.67	0.02	0.45
Perceived Control in Life	Resilience Skills and Beliefs	Resilience Resources	0.66	0.02	0.43
Perception of Things Going Smoothly	Resilience Skills and Beliefs	Resilience Resources	0.76	0.02	0.58
Perspective-taking	Resilience Skills and Beliefs	Resilience Resources	0.40	0.03	0.16
Positive Affect	Resilience Skills and Beliefs	Resilience Resources	0.70	0.02	0.49
Self-efficacy	Resilience Skills and Beliefs	Resilience Resources	0.63	0.02	0.40
Sleep Disturbances	Sleep	Resilience Resources	0.63	0.03	0.40
Sleep Latency	Sleep	Resilience Resources	0.53	0.03	0.29
Sleep Quality	Sleep	Resilience Resources	0.87	0.03	0.76
Support Satisfaction	Social Support	Resilience Resources	0.92	0.02	0.85
Trusted Support System	Social Support	Resilience Resources	0.80	0.02	0.64
**Note.** Items removed during EFA are not shown; see EFA Results section.					

Table S4. Phase 1 internal consistency statistics.

**Full WONE Index**	CR[a]	AVE[a]
Stress Subscale	0.87	0.60
Personal Stress Factor	0.86	0.67
Work Stress Factor	0.86	0.61
Burnout Factor	0.82	0.60

Resilience Resources Subscale	0.88	0.63
Resilience Skills and Beliefs Factor	0.89	0.42
Social Support Factor	0.85	0.74
Sleep Factor	0.74	0.43

CR, composite reliability; AVE, average variance extracted.
[a]CR and AVE estimates are not applicable to the full WONE Index as it is calculated as a composite of two separate confirmatory factor models, rather than being estimated as a latent factor within a single measurement model.

Table S5. Phase 1 HTMT matrix.

	Resilience Skills and Beliefs	Social Support	Sleep	Work Stress	Personal Stress	Burnout
Resilience Skills and Beliefs	–					
Social Support	0.66	–				
Sleep	0.50	0.30	–			
Work Stress	0.50	0.34	0.50	–		
Personal Stress	0.58	0.47	0.52	0.58	–	
Burnout	0.64	0.40	0.38	0.62	0.44	–

Table S6. Phase 1 correlations between WONE Index and established measures.

	Full WONE Index	WONE Stress Subscale	WONE Resilience Subscale	Perceived Stress Scale	CD-RISC Resilience Scale	Brief Resilience Scale	PROMIS-SF-8 Depressive Symptoms	GAD-7 Anxiety Symptoms	WHO-5 Well-being
Full WONE Index	–								
WONE Stress Subscale	-0.88[a]	–							
WONE Resilience Subscale	0.94[a]	-0.66[a]	–						

Perceived Stress Scale	-0.77[a]	0.66[a]	-0.74[a]	–					
CD-RISC Resilience Scale	0.70[a]	-0.52[a]	0.74[a]	-0.64[a]	–				
Brief Resilience Scale	0.67[a]	-0.51[a]	0.68[a]	-0.61[a]	0.77[a]	–			
PROMIS-SF-8 Depressive Symptoms	-0.77[a]	0.69[a]	-0.71[a]	0.77[a]	-0.59[a]	-0.58[a]	–		
GAD-7 Anxiety Symptoms	-0.72[a]	0.70[a]	-0.62[a]	0.70[a]	-0.52[a]	-0.54[a]	0.78[a]	–	
WHO-5 Well-being	0.79[a]	-0.66[a]	0.77[a]	-0.70[a]	0.56[a]	0.57[a]	-0.76[a]	-0.67[a]	–
[a]*P*<.001									

Modifications Added to Phase 2 Stress Load CFA Model

1) Covariances between first-order factors (Work Stress↔Burnout), reflecting conceptual overlap between immediate work stress and longer-term burnout experiences;
2) Covariances between the Burnout factor and error terms of work stress items (Burnout factor↔Stress item, Burnout factor↔Anxiousness item, Burnout factor↔Exhaustion item), indicating these work stress items capture variance relevant to both acute stress and developing burnout;
3) A covariance between the Personal Stress factor and the error term of the Exhaustion item (Personal Stress factor↔Exhaustion item), reflecting that exhaustion may be influenced by both work and personal stressors; and
4) Residual covariances between parallel items across stress domains (Anxiousness (Work) item↔Anxiousness (Personal) item; Overwhelm (Work) item↔Overwhelm (Personal) item; Cynicism item↔Productivity item).

Modifications Added to Phase 2 Resilience Resources CFA Model

1) Covariances between first-order factors (Emotion Regulation & Coping factor↔Purpose & Prosociality factor; Social factor↔Purpose & Prosociality factor), reflecting conceptual overlap among psychological resilience resources;
2) Covariances between factors and item error terms (Emotion Regulation & Coping factor↔Emotion Regulation item, Emotion Regulation & Coping factor↔Sleep Duration item; Social factor↔Ability to Bounce Back item; Purpose & Prosociality factor↔Emotion Regulation item, Purpose & Prosociality

factor↔Emotional Understanding item), indicating these items capture variance relevant to multiple resilience domains;

3) A covariance between the second-order Resilience Resources factor and the error term of the Meaning and Purpose item; and
4) Residual covariances between conceptually overlapping items (Compassion for Others item↔Consideration for Others item; Meaning and Purpose item↔Self Efficacy item; Acceptance item↔Emotional Understanding item; Emotion Regulation item↔Emotional Understanding item; Cognitive Flexibility item↔Adaptability item; Caffeine Intake item↔Caffeine Reliance item).

Table S7. HTRT results - Stress subscale

Factor	Personal Stress	Work Stress	Burnout
Personal Stress	–		
Work Stress	0.65	–	
Burnout	0.56	0.75	–

Table S8. HTRT results - Resilience Resources subscale

Factor	Dietary Intake	Emotion Regulation and Coping	Purpose and Prosociality	Physical Activity	Perseverative Thinking	Sleep	Social Connection
Dietary Intake	–						
Emotion Regulation and Coping	0.54	–					
Purpose and Prosociality	0.28	0.57	–				
Physical Activity	0.20	0.27	0.15	–			
Perseverative Thinking	0.45	0.63	0.33	0.23	–		
Sleep	0.48	0.64	0.35	0.25	0.56	–	
Social Connection	0.34	0.51	0.61	0.18	0.40	0.43	–

REFERENCES

1. MacCallum RC, Browne MW, Sugawara HM. Power analysis and determination of sample size for covariance structure modeling. Psychol Methods. 1996;1(2):130-149. [doi: 10.1037/1082-989X.1.2.130]

2. Hu L, Bentler PM. Cutoff criteria for fit indexes in covariance structure analysis: conventional criteria versus new alternatives. Struct Equ Modeling. Jan 1999;6(1):1-55. [doi: 10.1080/10705519909540118]

3. Bonett DG. Sample size requirements for testing and estimating coefficient alpha. J Educ Behav Stat. 2002;27(4):335-340. [doi: 10.3102/10769986027004335]

4. Zou GY. Sample size formulas for estimating intraclass correlation coefficients with precision and assurance. Stat Med. Dec 20, 2012;31(29):3972-3981. [doi: 10.1002/sim.5466] [Medline: 22764084]
